# Agreement of minimally invasive pulse wave analysis with pulmonary artery and transpulmonary thermodilution cardiac output measurements in perioperative and intensive care medicine: a systematic review and meta-analysis

**DOI:** 10.1016/j.bja.2026.04.045

**Published:** 2026-06-02

**Authors:** Moritz Flick, Dominik X. Müller, Alina Bergholz, Pawel Sierzputowski, Michael Eichlseder, Rami Saad, Gillis Greiwe, Karim Kouz, Alexander Hapfelmeier, Bernd Saugel

**Affiliations:** 1 Department of Anesthesiology, Center of Anesthesiology and Intensive Care Medicine, University Medical Center Hamburg-Eppendorf, Hamburg, Germany; 2 Division of Anaesthesiology and Intensive Care 1, Department of Anaesthesiology and Intensive Care, Medical University of Graz, Graz, Austria; 3 Outcomes Research Consortium®, Houston, Texas, USA; 4 Institute of AI and Informatics in Medicine, TUM School of Medicine and Health, Technical University of Munich, Munich, Germany; 5 Institute of General Practice and Health Services Research, TUM School of Medicine and Health, Technical University of Munich, Munich, Germany

**Keywords:** blood flow, cardiac output, haemodynamic monitoring, intensive care, pulse contour, stroke volume, thermodilution

## Abstract

**Background:**

Cardiac output monitoring is recommended for high-risk surgical patients and critically ill patients with circulatory shock. We performed a systematic review and meta-analysis of clinical studies published since 2010 that compared minimally invasive pulse wave analysis-derived cardiac output or cardiac index measurements with reference measurements by pulmonary artery thermodilution or transpulmonary thermodilution in adult surgical or critically ill patients.

**Methods:**

In a random-effects meta-analysis, we calculated pooled estimates of the percentage error, mean difference and standard deviation, and 95% limits of agreement separately for studies reporting cardiac output or cardiac index. Subgroup analyses were performed by patient population and test device.

**Results:**

We included 92 studies divided into 113 data sets with a total of 3111 patients. For 71 data sets reporting cardiac output, the pooled percentage error (95% confidence interval [95% CI]) was 44.0% (38.2%–49.8%) with a mean difference (standard deviation) of -0.1 (1.3) L min^−1^ with 95% limits of agreement of -2.6 to 2.4 L min^−1^ (I^2^=9.8%). For 42 data sets reporting cardiac index, the pooled percentage error was 49.1% (40.6%–57.6%) with a mean difference of -0.1 (0.9) L min^−1^ m^−2^ with 95% limits of agreement of -1.8 to 1.6 L min^−1^ m^−2^ (I^2^=7.9%). The percentage error varied substantially across patient populations and devices. Overall risk of bias was low.

**Conclusions:**

The pooled percentage error between minimally invasive pulse wave analysis-derived cardiac output measurements of 44.0% (cardiac output) and 49.1% (cardiac index) in adult surgical or critically ill patients exceeds the 30% threshold for clinically acceptable agreement. However, the percentage error varied depending on the patient population and device used.

Trial Registration. PROSPERO (CRD420251090806; submitted July 17, 2025).

**Editor’s key points**

• Minimally invasive pulse wave analysis is widely used to monitor cardiac output in surgical and critically ill patients.
• This systematic review and meta-analysis assessed the agreement between cardiac output and cardiac index measured by minimally invasive pulse wave analysis and reference thermodilution methods in surgical and critically ill patients.
• The pooled percentage errors of 44.0% (cardiac output) and 49.1% (cardiac index) exceed the 30% threshold for clinically acceptable agreement. The percentage error varied depending on the patient population and device.

Cardiac output monitoring is recommended for high-risk surgical patients^1^ and critically ill patients with circulatory shock.^2^ Measuring cardiac output can help clinicians understand the underlying causes of hypotension,^3^^,^^4^ diagnose the type of shock,^5^^,^^6^ and titrate fluids, vasopressors, and inotropes.^2^^,^^6^ Pulmonary artery thermodilution^7^^,^^8^ and transpulmonary thermodilution^9^ are considered clinical reference methods for cardiac output measurements. However, over the last decades, less invasive cardiac output monitoring methods have become available,^10^, ^11^, ^12^ including minimally invasive pulse wave analysis, which allows measuring cardiac output by mathematically analysing the arterial pressure waveform recorded with an arterial catheter.^13^, ^14^, ^15^

Minimally invasive pulse wave analysis is commonly used to measure cardiac output,^15^, ^16^, ^17^ but its measurement performance can be influenced by rapid changes in blood pressure and systemic vascular resistance, e.g. because of vasoplegia with venodilation or hypovolaemia.^14^ Further, the measurement performance may vary depending on the specific pulse wave analysis algorithm used in the different available devices.^18^ The measurement performance of minimally invasive pulse wave analysis, therefore, is a matter of ongoing debate.^14^

In 2010, a meta-analysis of 24 studies reported a pooled percentage error of 41.3% between cardiac output measurements with pulse wave analysis and reference measurements with thermodilution methods — with percentage errors reported in individual studies ranging from 20% to 70%.^19^ Since then, existing pulse wave analysis algorithms have been updated, and new algorithms have been introduced. Consequently, the level of agreement between currently available minimally invasive pulse wave analysis devices and reference thermodilution methods is unclear.

We therefore aimed to assess the agreement of minimally invasive pulse wave analysis cardiac output measurements with pulmonary artery and transpulmonary thermodilution in perioperative and intensive care medicine in an updated meta-analysis. We performed a systematic review and meta-analysis of clinical studies published since 2010 that compared minimally invasive pulse wave analysis-derived cardiac output or cardiac index measurements with reference measurements by pulmonary artery thermodilution or transpulmonary thermodilution in adult surgical or critically ill patients. We analysed the mean difference, the 95% limits of agreement (95% LoA), and the percentage error.

## Method

### Study design

This systematic review and meta-analysis was conducted according to the Preferred Reporting Items for Systematic Reviews and Meta-Analyses (PRISMA) guidelines.^20^ This systematic review and meta-analysis was prospectively registered in the International Prospective Register of Systematic Reviews (PROSPERO; registration number CRD420251090806; submitted July 17, 2025). We also considered relevant aspects of the ‘Statistical Analysis and Reporting of Cardiac Output Method Comparison Studies’ (COMPARE) statement.^21^

We conducted a systematic search of the electronic database PubMed. Upon reviewer’s request, we expanded the search to include the Web of Science and Cochrane databases. The complete search strategies are provided in Supplementary material S1.

The eligibility criteria for this meta-analysis were defined according to the PECOS framework (Population, Exposure, Comparator, Outcomes, and Study design). Regarding populations, we included studies with adult (≥18 yr) surgical or critically ill patients. We did not include studies with paediatric patients or healthy volunteers. Concerning the exposure, we only included studies that investigated internally calibrated or uncalibrated minimally invasive pulse wave analysis-derived cardiac output or cardiac index measurements (test method).^13^, ^14^, ^15^ We excluded studies that investigated externally calibrated minimally invasive pulse wave analysis or invasive pulse wave analysis (that is, by definition, calibrated to an external cardiac output value, e.g. from transpulmonary thermodilution).^13^, ^14^, ^15^ With respect to the comparator, we only included studies that compared the test method with pulmonary artery thermodilution or transpulmonary thermodilution (reference method). We excluded studies with other reference methods (e.g. echocardiography and the Fick method). For the outcomes, we only included studies that reported the mean difference (or the mean values of both the test method and the reference method); the standard deviation of the differences or the 95% LoA, or the percentage error between minimally invasive pulse wave analysis-derived cardiac output/cardiac index measurements and reference measurements by pulmonary artery thermodilution or transpulmonary thermodilution. We excluded studies that did not perform Bland–Altman analyses or that reported insufficient data for quantitative synthesis. Finally, we included original method comparison studies (prospective and retrospective) published in English between January 1, 2010, and July 14, 2025. We excluded poster abstracts and case reports.

### Study selection

**Fig 1:**
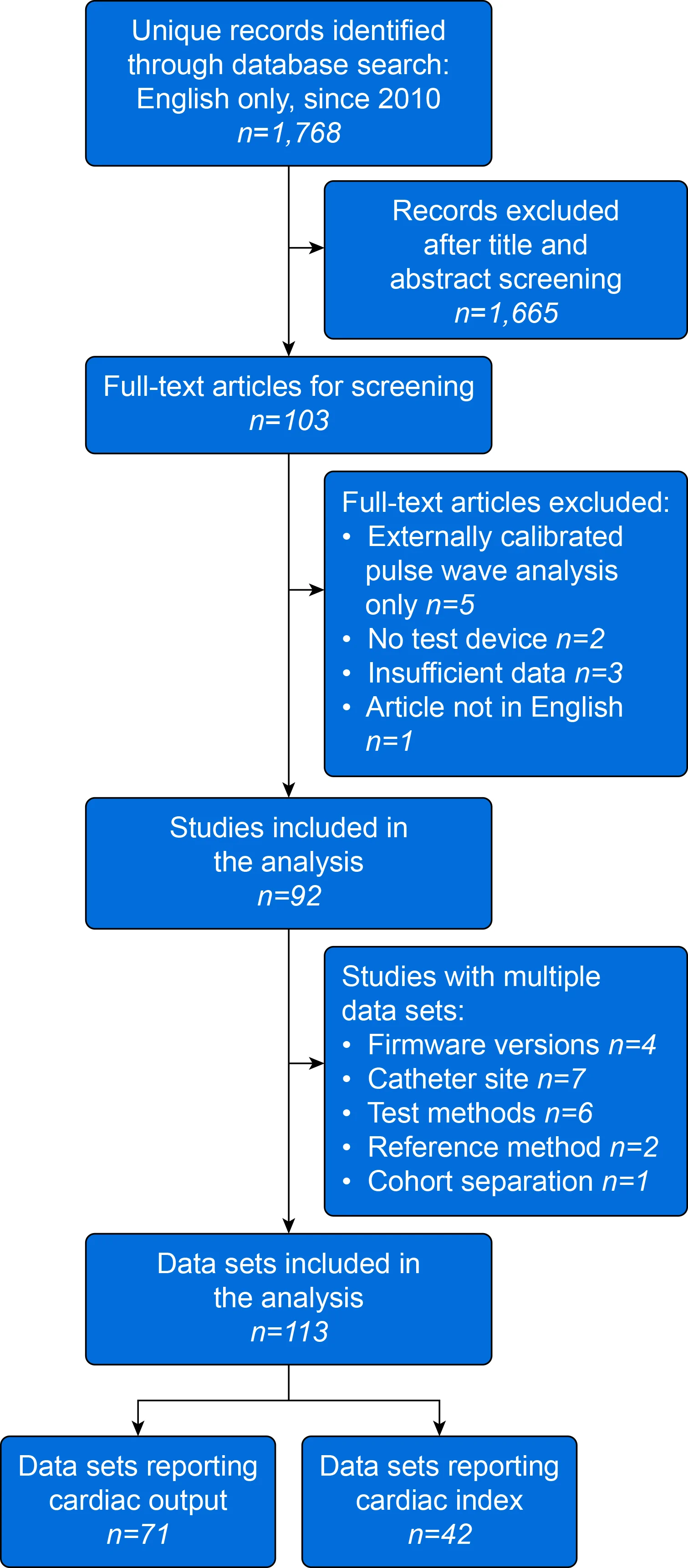
Flowchart of the literature search with the number of the included studies based on the PRISMA statement.

Two reviewers (MF, DXM) independently screened titles and abstracts using a web-based systematic review management platform (Rayyan; Qatar Computing Research Institute, Hamad Bin Khalifa University, Doha, Qatar). In addition, the full-text articles were accessed to determine the eligibility of studies. Disagreements were resolved through discussion and with consultation of a third reviewer (BS) when necessary. The study selection process was documented using a PRISMA flow diagram (Fig. 1).

### Data collection process

Four investigators (DXM, AB, PS, and ME) independently extracted data from the included studies using an electronic spreadsheet (Excel; Microsoft Corporation, Redmond, WA, USA). Data from each study were extracted independently from three of these investigators. The extracted data were reviewed by another investigator (MF) for consistency, and any discrepancies were resolved by discussion of the original data.

The extracted data included the mean measurements of the test and reference method as well as the results of comparative statistics, i.e. the mean difference, the standard deviation of the differences, the 95% LoA, and the percentage error between minimally invasive pulse wave analysis-derived cardiac output or cardiac index measurements and reference measurements by pulmonary artery thermodilution or transpulmonary thermodilution. In addition, we systematically extracted details regarding the study setting, patient cohort, and measurement time points.

### Risk of bias in individual studies

The quality of the included studies was assessed using the Quality Assessment of Diagnostic Accuracy Studies (QUADAS-2) tool,^22^ with the assessment questions adapted to cardiac output method comparison studies (Supplementary material S2), as described previously.^23^, ^24^, ^25^ The QUADAS-2 framework comprises four domains for evaluating the risk of bias and three domains for evaluating concerns regarding applicability. Each domain contains signalling questions, which are answered as ‘yes’, ‘no’, or ‘unclear’. The questionnaire used in the present review is provided in the Supplementary material S2. For each domain, the risk of bias and applicability concerns were classified as ‘low’, ‘high’, or ‘unclear’ based on the responses to the signalling questions. Per the reviewer’s request, we also assessed the methods and result report of the included studies using a checklist with the following domains: measurement protocol described, appropriate reference method, redefined acceptable error (e.g. 30%), sample size justification, normality checked, Bland–Altman correctly applied, mean difference 95% LoA reported, percentage error reported, confidence intervals reported, repeated measures handled, and proportional bias assessed.^21^^,^^26^ Each domain was classified as ‘yes’, ‘no’, or ‘unclear’. Three out of four investigators (DXM, AB, PS, or MF) independently assessed the risk of bias of each included study. Any discrepancies were resolved by re-examination of the original publication and discussion among the investigators.

### Synthesis of results

Several data pre-processing steps were performed to provide suitable data for meta-analysis. In two studies, the mean difference between minimally invasive pulse wave analysis-derived cardiac output or cardiac index measurements and reference measurements by pulmonary artery thermodilution or transpulmonary thermodilution was not reported and was thus imputed by the difference between mean measurements reported for each method. Missing information about the standard deviation of the differences was derived from the reported 95% LoA in 64 (44%) data sets or from the reported percentage error in three (2%) data sets. In 26 of these data sets, it was not reported whether the quantiles of the normal distribution or the t-distribution had been used to calculate the 95% LoA or percentage error. We then applied the quantile of the normal distribution (i.e. 1.96) in our computations to deduce a conservative (i.e. larger) estimate of the standard deviation of the differences.

Some studies reported comparative measurements at different procedural times. Using the number of subjects included in the respective analyses as weights, this multiplicity was resolved by calculating weighted means of the mean differences and variances of the differences. These weighted means and the average sample size were subsequently used in the meta-analysis.

We finally applied the random-effects meta-analysis of Bland–Altman studies based on a limits of agreement approach with robust variance estimation.^27^ This approach was done separately for studies reporting cardiac output and cardiac index, as their absolute values are not comparable in magnitude and cannot be meaningfully pooled in a single meta-analysis. Converting all values into one variable was not feasible without access to individual patient data. The random-effects meta-analysis estimates the mean difference δ, the within-study variation $\sigma^{2}$ and the between-study variation $\tau^{2}$, whereas the term ‘study’ refers to a Bland-Altman analysis. These estimates were used to deduce the $LoA=\delta \pm 2\sqrt{\sigma^{2}+\tau^{2}}$ and the percentage error $=2\sqrt{\sigma^{2}+\tau^{2}}/\mu_{ref}$. Here, $\mu_{ref}$ is the mean measurement of the reference method and was estimated through another meta-analysis of single means, using the numbers of subjects of the included Bland-Altman-analyses as conservative sample sizes.^28^ The 95% confidence interval (95% CI) of the percentage error was calculated as $PE\pm 2\sqrt{Var\left(PE\right)}$, where $Var\left(PE\right)=Var\left(2\sqrt{\sigma^{2}+\tau^{2}}/\mu_{ref}\right)={\left(1/\mu_{ref}\right)}^{2}Var\left(2\sqrt{\sigma^{2}+\tau^{2}}\right)+{\left(2\sqrt{\sigma^{2}+\tau^{2}}/{\mu_{ref}}^{2}\right)}^{2}Var\left(\mu_{ref}\right)$ was estimated by the delta method and by plugging in the estimates obtained from the meta-analyses described above. Between-study heterogeneity was assessed by $I^{2}=\tau^{2}/\left(\tau^{2}+\sigma^{2}\right)$. Differences between summary measures were not explicitly analysed but can be deduced from the point estimates and respective 95% CIs.

Upon reviewer’s request, we performed a sensitivity analysis restricted to data sets with low risk of bias according to the QUADAS-2 tool.

### Subgroup analyses

We performed subgroup analyses restricted to certain patient groups: cardiac surgery patients (intra- and postoperative), critically ill patients (medical or surgical patients treated in the intensive care unit), liver transplant patients, noncardiac surgery patients, and mixed patient cohorts.

We also performed subgroup analyses restricted to the following test devices: FloTrac (Edwards Lifesciences; Irvine, CA, USA), ArgosCO (Retia Medical Systems; White Plains, NY, USA), LiDCOrapid (Masimo; Irvine, CA, USA), MostCare (Vygon; Écouen, France), and ProAQT (Getinge; Gothenburg, Sweden).

### Statistical software

We used R version 4.1.3 (The R Foundation for Statistical Computing, Vienna, Austria) to perform all statistical analyses. The meta-analysis of Bland–Altman studies based on a limits of agreement approach was implemented through R-Code provided by supplementary material of the original publication.^27^ The meta-analysis of single means was implemented by use of the R-package ‘meta’.^29^

## Results

### Study selection

After removal of duplicates, we identified 1768 records from the initial electronic database search (Fig. 1). We excluded 1665 records after title and abstract screening. Full-text screening of the remaining 103 records identified 92 observational method comparison studies that fulfilled our predefined criteria (Supplementary material S3).^30^, ^31^, ^32^, ^33^, ^34^, ^35^, ^36^, ^37^, ^38^, ^39^, ^40^, ^41^, ^42^, ^43^, ^44^, ^45^, ^46^, ^47^, ^48^, ^49^, ^50^, ^51^, ^52^, ^53^, ^54^, ^55^, ^56^, ^57^, ^58^, ^59^, ^60^, ^61^, ^62^, ^63^, ^64^, ^65^, ^66^, ^67^, ^68^, ^69^, ^70^, ^71^, ^72^, ^73^, ^74^, ^75^, ^76^, ^77^, ^78^, ^79^, ^80^, ^81^, ^82^, ^83^, ^84^, ^85^, ^86^, ^87^, ^88^, ^89^, ^90^, ^91^, ^92^, ^93^, ^94^, ^95^, ^96^, ^97^, ^98^, ^99^, ^100^, ^101^, ^102^, ^103^, ^104^, ^105^, ^106^, ^107^, ^108^, ^109^, ^110^, ^111^, ^112^, ^113^, ^114^, ^115^, ^116^, ^117^, ^118^, ^119^, ^120^, ^121^ Of these 92 studies, 89 were prospective^30^, ^31^, ^32^, ^33^, ^34^, ^35^, ^36^, ^37^, ^38^, ^39^, ^40^, ^41^, ^42^, ^43^, ^44^, ^45^, ^46^, ^47^, ^48^, ^49^, ^50^, ^51^, ^52^, ^53^, ^54^, ^55^, ^56^, ^57^, ^58^, ^59^, ^60^, ^61^, ^62^, ^63^, ^64^, ^65^, ^66^, ^67^, ^68^, ^69^, ^70^, ^71^, ^72^, ^73^, ^74^, ^75^, ^76^, ^77^, ^78^, ^79^, ^80^, ^81^, ^82^, ^83^, ^84^, ^85^, ^86^, ^87^, ^88^, ^89^, ^90^, ^91^, ^92^, ^93^, ^94^, ^95^, ^96^, ^97^, ^98^, ^99^, ^100^, ^101^, ^102^, ^103^, ^104^, ^105^, ^106^, ^107^, ^108^, ^109^, ^110^, ^111^, ^112^, ^113^, ^114^, ^115^, ^116^, ^117^, ^118^ and three were retrospective.^119^, ^120^, ^121^ Twenty studies were divided into 41 distinct data sets because they separately reported results for multiple firmware versions of a pulse wave analysis test method,^31^^,^^41^^,^^47^^,^^51^ different arterial catheter sites (radial and femoral arterial catheters),^32^^,^^36^^,^^39^^,^^42^^,^^59^^,^^88^^,^^92^ multiple pulse wave analysis test methods,^54^^,^^79^^,^^90^^,^^95^^,^^104^^,^^115^ multiple reference methods,^70^^,^^72^ or for patients with and without atrial fibrillation.^66^ Thus, a total of 113 data sets with 3111 patients were included in the final analysis; the median (25th to 75th percentile) number of patients per data set was 22 (19–32).

### Study characteristics

Of the analysed 113 data sets (Table 1), 71 reported cardiac output with a total of 1923 patients and 42 reported cardiac index with a total of 1188 patients. The median number of patients per data set was 22 (18–35) for cardiac output and 22 (20–31) for cardiac index. Fifty-six data sets reported measurements in cardiac surgery patients, 35 in critically ill patients, 14 in liver transplant patients, six in noncardiac surgery patients, and two in mixed patient populations.

**Table 1:** Study characteristics. Capital letters (A, B, C) indicate separation into multiple data sets; # insufficient data, imputed for inclusion in the meta-analysis. PATD, pulmonary artery thermodilution; TPTD, transpulmonary thermodilution; int, intermittent; cont, continuous; SD, standard deviation; LLoA, lower limit of agreement; ULoA, upper limit of agreement; PE, percentage error; ns, not specified.

a) Studies reporting cardiac output.													
First author	Year	Journal	Device	Firmware-version	Reference	Int./ Cont.	Setting	*n*	Mean difference (L min^−1^)	SD (L min^−1^)	LLoA (L min^−1^)	ULoA (L min^−1^)	PE (%)
C. Slagt^31^ – A	2010	*Eur J Anaesthesiol*	FloTrac	1^st^ Gen (1.07)	PATD	int	critically ill	4	-1.60	1.60	-4.80	1.60	48.0
C. Slagt – B	2010	*Eur J Anaesthesiol*	FloTrac	1^st^ Gen (1.10)	PATD	int	critically ill	5	-1.20	1.10	-3.40	1.00	32.0
CK. Hofer^32^ – A	2010	*J Cardiothoracic Vasc Anesth*	FloTrac	1^st^ Gen (1.07)	PATD	int	cardiac surgery	26	0.27	0.98	-1.68	2.22	39.4
CK. Hofer – B	2010	*J Cardiothoracic Vasc Anesth*	FloTrac	1^st^ Gen (1.07)	PATD	int	cardiac surgery	26	0.20	1.05	-1.89	2.29	42.3
JB. Hamm^33^	2010	*Anaesth Intensive Care*	FloTrac	1^st^ Gen (1.07)	PATD	cont	cardiac surgery	9	-0.09	1.18	-2.45	2.27	48.0
M. Cecconi^34^	2010	*Minerva Anestesiol*	FloTrac	1^st^ Gen (1.03)	PATD	int	critically ill	29	-1.10	1.89	-4.88	2.68	55.0
M. Hadian^35^	2010	*Critical Care*	FloTrac	2^nd^ Gen	PATD	mixed	cardiac surgery	17	0.43	1.72	-3.01	3.87	59.0
S. Schramm^36^ – A	2010	*J Cardiothorac Vasc Anesth*	FloTrac	1^st^ Gen (1.07)	PATD	int	cardiac surgery	20	-0.35	1.88	-4.11	3.41	76.0
S. Schramm – B	2010	*J Cardiothorac Vasc Anesth*	FloTrac	1^st^ Gen (1.07)	PATD	int	cardiac surgery	20	0.11	1.80	-3.49	3.71	69.0
SF. Boettger^37^	2010	*Med Sci Monit*	FloTrac	ns	TPTD	int	critically ill	19	-0.72	1.47	-3.67	2.23	49.1
YB. Jeong^40^	2010	*J Cardiothorac Vasc Anesth*	FloTrac	1^st^ Gen (1.10)	PATD	cont	cardiac surgery	28	0.23	1.11	-2.00	2.46	57.0
D. De Backer^41^ – A	2011	*Intensive Care Med*	FloTrac	1^st^ Gen (1.14)	PATD	int	critically ill	58	-1.00	1.07	-3.15	1.15	28.6
D. De Backer – B	2011	*Intensive Care Med*	FloTrac	1^st^ Gen (1.14)	PATD	int	critically ill	58	-0.20	1.14	-2.48	2.08	30.4
E. Saraceni^42^ – A	2011	*Br J Anaesth*	FloTrac	1^st^ Gen (1.07)	PATD	int	critically ill	15	0.07	1.87	-3.67	3.81	57.6
E. Saraceni – B	2011	*Br J Anaesth*	FloTrac	1^st^ Gen (1.10)	PATD	int	critically ill	6	1.04	1.10	-1.17	3.25	34.4
EK. Junttila^43^	2011	*Br J Anaesth*	FloTrac	1^st^ Gen (1.10/1.17)	PATD	int	critically ill	16	-1.50	2.00	-5.50	2.50	58.0
F. Franchi^44^	2011	*Br J Anaesth*	MostCare	ns	PATD	int	critically ill	30	0.26	0.98	-1.70	2.22	25.0
H. Paarmann^46^	2011	*Br J Anaesth*	MostCare	ns	PATD	int	cardiac surgery	23	0.00	2.32	-4.63	4.63	87.0
K. Akiyoshi^47^ – A	2011	*J Anesth*	FloTrac	1^st^ Gen (1.0)	PATD	int	liver transplant	20	-1.73	1.09	-3.91	0.45	30.3
K. Akiyoshi – B	2011	*J Anesth*	FloTrac	3^rd^ Gen (3.0)	PATD	int	liver transplant	20	-0.89	1.35	-3.59	1.81	37.5
L. Vetrugno^48^	2011	*J Cardiothorac Vasc Anesth*	FloTrac	1^st^ Gen (1.10)	PATD	int	cardiac surgery	15	0.50	0.86	-1.22	2.22	37.0
M. Haenggi^49^	2011	*Resuscitation*	FloTrac	1^st^ Gen (1.07)	PATD	cont	critically ill	8	-0.50	0.69	-1.88	0.88	29.0
S. Metzelder^51^ – A	2011	*Br J Anaesth*	FloTrac	2^nd^ Gen	TPTD	int	critically ill	10	-0.90	1.33	-3.55	1.75	29.6
S. Metzelder – B	2011	*Br J Anaesth*	FloTrac	3^rd^ Gen	TPTD	int	critically ill	14	-1.00	1.17	-3.35	1.35	27.9
S. Scolletta^52^	2011	*Anesth Analg*	MostCare	ns	PATD	int	cardiac surgery	15	0.20	0.50	-0.81	1.21	24.0
S. Scolletta^53^	2011	*Interact Cardiovasc Thorac Surg*	MostCare	ns	PATD	cont	critically ill	12	0.04	0.19	-0.34	0.42	7.3
BC. Su^56^	2012	*Transplant Proc*	FloTrac	3^rd^ Gen	PATD	cont	liver transplant	28	-0.80	2.45	-5.70	4.10	75.0
JK. Lu^57^	2012	*Chin Med J (Engl)*	FloTrac	1^st^ Gen (1.07)	TPTD	int	cardiac surgery	50	-0.02	0.35	-0.72	0.68	15.8
S. Vasdev^59^ – A	2012	*J Clin Monit Comput*	FloTrac	3^rd^ Gen (3.02)	PATD	int	cardiac surgery	38	-0.14	0.56	-1.26	0.98	22.0
S. Vasdev – B	2012	*J Clin Monit Comput*	FloTrac	3^rd^ Gen (3.02)	PATD	int	cardiac surgery	38	0.13	0.50	-0.87	1.13	20.0
YF. Tsai^62^	2012	*Transplant Proc*	FloTrac	3^rd^ Gen	PATD	int	liver transplant	20	-0.22	1.70	-3.63	3.19	54.9
C. Slagt^63^	2013	*J Clin Monit Comput*	FloTrac	3^rd^ Gen (3.02)	PATD	int	critically ill	19	-1.70	2.40	-6.50	3.10	53.0
K. Suehiro^65^	2013	*Br J Anaesth*	FloTrac	3^rd^ Gen (3.02)	PATD	int	cardiac surgery	40	0.08	0.93	-1.78	1.93	45.5
O. Desebbe^67^	2013	*J Cardiothorac Vasc Anesth*	FloTrac	3^rd^ Gen (3.01)	PATD	int	cardiac surgery	25	-0.21	1.37	-2.95	2.53	66.5
Y. Sotomi^69^	2013	*J Clin Monit Comput*	FloTrac	3^rd^ Gen (3.02)	PATD	cont	cardiac surgery	50	0.20	1.30	-2.40	2.80	54.4
A. Donati^70^– A	2014	*J Crit Care*	MostCare	ns	TPTD	int	critically ill	32	-0.67	0.89	-2.45	1.11	28.0
A. Donati – B	2014	*J Crit Care*	MostCare	ns	PATD	cont	cardiac surgery	16	-0.22	0.55	-1.32	0.88	25.0
MG. Costa^72^ – A	2014	*J Cardiothorac Vasc Anesth*	LiDCOrapid	ns	PATD	int	liver transplant	20	0.10	1.25	-2.39	2.59	39.2
MG. Costa – B	2014	*J Cardiothorac Vasc Anesth*	LiDCOrapid	ns	PATD	cont	liver transplant	20	-0.79	1.47	-3.73	2.15	34.6
P. Wacharasint^73^	2014	*J Med Assoc Thai*	FloTrac	3^rd^ Gen (3.06)	PATD	int	cardiac surgery	50	0.70	1.63	-2.56	3.96	#
S. Gopal^74^	2014	*Minerva Anestesiol*	MostCare	ns	PATD	int	critically ill	20	-0.31	1.97	-4.25	3.63	62.5
C. Slagt^75^	2015	*Eur J Anaesthesiol*	FloTrac	3^rd^ Gen (3.02)	TPTD	int	critically ill	20	-0.86	1.85	-4.56	2.84	48.0
K. Suehiro^76^	2015	*J Cardiothorac Vasc Anesth*	FloTrac	4^th^ Gen (4.0)	PATD	int	cardiac surgery	23	0.66	0.95	-1.23	2.55	55.4
LJ. Montenij^77^	2015	*Eur J Anaesthesiol*	FloTrac	3^rd^ Gen (3.02)	PATD	int	cardiac surgery	22	-0.54	1.73	-4.00	2.92	58.0
BF. Shih^80^	2016	*Transplant Proc*	FloTrac	4^th^ Gen (4.0)	PATD	cont	liver transplant	40	-0.51	1.64	-3.79	2.77	42.2
DS. Eiferman^81^	2016	*J Intensive Care Med*	FloTrac	ns	PATD	cont	critically ill	14	-0.68	0.25	-1.17	-0.18	#
LJ. Montenij^83^	2016	*J Cardiothorac Vasc Anesth*	FloTrac	3^rd^ Gen (3.02)	PATD	int	cardiac surgery	22	0.69	1.12	-1.54	2.92	55.0
MT. Ganter^84^	2016	*J Clin Monit Comput*	FloTrac	3^rd^ Gen (3.02)	PATD	int	critically ill	47	0.00	1.09	-2.18	2.18	34.5
T. Terada^86^	2016	*J Clin Monit Comput*	FloTrac	3^rd^ Gen	PATD	int	noncardiac surgery	15	0.04	1.37	-2.70	2.78	42.5
YJ. Cho^87^	2016	*J Clin Monit Comput*	FloTrac	4^th^ Gen (4.0)	PATD	int	cardiac surgery	20	-0.05	0.72	-1.50	1.40	33.8
A. van Drumpt^88^ – A	2017	*BMC Anesthesiol*	ProAQT	ns	TPTD	int	cardiac surgery	25	0.31	1.42	-2.53	3.15	49.0
A. van Drumpt – B	2017	*BMC Anesthesiol*	ProAQT	ns	TPTD	int	cardiac surgery	25	0.57	1.37	-2.17	3.31	46.0
O. Antal^93^	2017	*Rom J Anaesth Intensive Care*	FloTrac	3^rd^ Gen (3.06)	TPTD	int	critically ill	4	0.30	1.38	-2.46	3.06	82.0
B. Lamia^94^	2018	*J Clin Monit Comput*	FloTrac	3^rd^ Gen (3.0)	PATD	int	cardiac surgery	21	0.40	1.17	-1.95	2.75	40.0
S. Lin^96^	2018	*J Clin Monit Comput*	FloTrac	4^th^ Gen	PATD	cont	cardiac surgery	32	0.32	1.22	-2.11	2.75	56.8
M. Geisen^95^– A	2018	*J Cardiothorac Vasc Anesth*	FloTrac	3^rd^ Gen (3.02)	TPTD	int	cardiac surgery	22	-0.54	1.52	-3.59	2.51	51.4
M. Geisen – B	2018	*J Cardiothorac Vasc Anesth*	LiDCOrapid	ns	TPTD	int	cardiac surgery	22	-0.17	1.75	-3.68	3.34	57.2
A. Eisenried^97^	2019	*J Cardiothorac Vasc Anesth*	FloTrac	4^th^ Gen	PATD	cont	cardiac surgery	20	0.30	1.71	-3.12	3.72	64.5
Y. Kusaka^99^	2019	*J Cardiothorac Vasc Anesth*	FloTrac	4^th^ Gen (4.0)	PATD	int	cardiac surgery	30	0.41	0.72	-1.03	1.85	37.1
B. Saugel^101^	2020	*J Clin Monit Comput*	ArgosCO	ns	PATD	int	cardiac surgery	58	0.20	1.14	-2.08	2.48	50.8
G. Greiwe^102^	2020	*Eur J Anaesthesiol*	MostCare	ns	PATD	int	cardiac surgery	41	-0.08	0.74	-1.56	1.40	29.8
G. Greiwe^103^	2020	*J Clin Monit Comput*	ArgosCO	ns	PATD	int	cardiac surgery	31	-0.08	1.10	-2.28	2.12	40.7
AJ. Milam^105^	2021	*J Cardiothorac Vasc Anesth*	FloTrac	ns	PATD	int	cardiac surgery	44	0.11	1.20	-2.29	2.51	49.0
P. Persona^106^	2021	*J Clin Med*	MostCare	ns	TPTD	int	critically ill	40	-0.11	0.67	-1.46	1.23	24.8
R. Mukkamala^107^	2021	*BMC Anesthesiol*	FloTrac	3^rd^ Gen (3.02)	PATD	int	cardiac surgery	58	0.94	1.24	-1.55	3.43	60.3
C. Oh^120^	2022	*J Clin Med*	FloTrac	4^th^ Gen	PATD	cont	cardiac surgery	30	0.94	1.38	-1.83	3.71	66.1
K. Halemani^121^	2022	*Turk J Anaesthesiol Reanim*	FloTrac	3^rd^ Gen (3.02)	PATD	cont	liver transplant	58	-0.44	2.23	-4.90	4.02	56.8
Y. Murata^110^	2022	*Sci Rep*	FloTrac	4^th^ Gen (4.00)	PATD	cont	liver transplant	20	-1.62	2.37	-6.36	3.12	54.4
AK. Khanna^112^	2023	*J Clin Monit Comput*	ArgosCO	ns	PATD	cont	cardiac surgery	79	0.04	1.04	-2.04	2.12	38.2
NH. Wu^113^	2023	*Heart Vessels*	FloTrac	4^th^ Gen (4.0)	PATD	cont	cardiac surgery	10	0.54	1.51	-2.48	3.56	51.2
C. Dinesen^118^	2025	*Acta Anaesthesiol Scand*	FloTrac	4^th^ Gen	PATD	int	cardiac surgery	41	0.33	0.88	-1.44	2.10	40.2
**Summary**								**1923**	-0.10	1.26	-2.62	2.42	**44.0**

b) Studies reporting cardiac index.													
First author	Year	Journal	Device	Firmware-version	Reference	Int./ Cont.	Setting	*n*	Mean difference (L min^−1^ m^−2^)	SD (L min^−1^ m^−2^)	LLoA (L min^−1^ m^−2^)	ULoA (L min^−1^ m^−2^)	PE (%)
A. Zangrillo^30^	2010	*J Cardiothorac Vasc Anesth*	MostCare	ns	PATD	int	cardiac surgery	28	-0.07	0.41	-0.89	0.75	30.0
V. Krejci^38^	2010	*Liver Transpl*	FloTrac	1^st^ Gen (1.10)	PATD	int	liver transplant	19	-1.78	1.39	-4.56	1.00	68.5
X. Monnet^39^ – A	2010	*Critical Care*	FloTrac	1^st^ Gen (1.10)	TPTD	int	critically ill	80	-0.09	0.94	-1.97	1.79	61.0
X. Monnet – B	2010	*Critical Care*	FloTrac	1^st^ Gen (1.10)	TPTD	int	critically ill	20	-0.47	0.84	-2.15	1.21	57.0
G. Biancofiore^45^	2011	*Anesth Analg*	FloTrac	3^rd^ Gen (3.02)	PATD	int	noncardiac surgery	21	-0.38	1.17	-2.71	1.95	52.0
O. Broch^50^	2011	*Critical Care*	LiDCOrapid	ns	TPTD	int	cardiac surgery	42	0.18	0.97	-1.76	2.13	69.3
TD. Phan^54^ – A	2011	*Anaesth Intensive Care*	FloTrac	1^st^ + 3^rd^ Gen (1.14/3.0)	PATD	int	cardiac surgery	15	-0.21	0.68	-1.57	1.15	47.6
TD. Phan – B	2011	*Anaesth Intensive Care*	LiDCOrapid	ns	PATD	int	cardiac surgery	15	0.11	0.78	-1.45	1.67	54.2
YY. Jo^55^	2011	*Korean J Anesthesiol*	FloTrac	1^st^ Gen (1.07)	PATD	cont	cardiac surgery	50	-0.07	0.34	-0.74	0.60	26.9
O. Broch^58^	2012	*Scientific World Journal*	FloTrac	1^st^ Gen (1.07)	TPTD	int	cardiac surgery	47	0.02	0.35	-0.68	0.73	56.5
T. Mutoh^60^	2012	*Surg Neurol Int*	FloTrac	3^rd^ Gen (3.02)	TPTD	int	critically ill	20	-0.33	0.26	-0.85	0.19	14.9
X. Monnet^61^	2012	*Br J Anaesth*	FloTrac	3^rd^ Gen	TPTD	int	critically ill	20	0.26	0.94	-1.62	2.14	54.0
J. Grensemann^64^	2013	*Anesth Analg*	FloTrac	1^st^ Gen (1.07)	TPTD	int	critically ill	16	-0.23	0.86	-1.95	1.49	50.9
L. Barile^66^ – A	2013	*J Cardiothorac Vasc Anesth*	MostCare	ns	PATD	int	cardiac surgery	48	-0.05	0.40	-0.84	0.74	29.0
L. Barile – B	2013	*J Cardiothorac Vasc Anesth*	MostCare	ns	PATD	int	cardiac surgery	11	-0.20	0.88	-1.97	1.57	69.0
S. Marqué^68^	2013	*J Clin Monit Comput*	FloTrac	3^rd^ Gen	PATD	cont	critically ill	18	-0.10	1.04	-2.19	1.99	64.0
AA. Smetkin^71^	2014	*Br J Anaesth*	ProAQT	ns	TPTD	int	cardiac surgery	20	-0.14	0.42	-0.98	0.70	31.0
O. Broch^78^	2015	*BMC Anesthesiol*	ProAQT	ns	TPTD	int	cardiac surgery	32	0.57	0.81	-1.04	2.18	63.1
X. Monnet^79^ – A	2015	*Br J Anaesth*	FloTrac	3^rd^ Gen	TPTD	int	critically ill	60	0.50	0.95	-1.40	2.40	59.0
X. Monnet – B	2015	*Br J Anaesth*	ProAQT	ns	TPTD	int	critically ill	60	-0.10	0.65	-1.40	1.20	40.0
X. Monnet – C	2015	*Br J Anaesth*	ProAQT	ns	TPTD	int	critically ill	60	-0.10	0.70	-1.50	1.30	39.0
J. Grensemann^82^	2016	*Anaesth Intensive Care*	ProAQT	ns	TPTD	int	critically ill	21	0.25	0.92	-1.59	2.09	51.2
R. Tomasi^85^	2016	*J Clin Monit Comput*	FloTrac	3^rd^ Gen (3.02)	PATD	cont	noncardiac surgery	13	-0.55	0.90	-2.35	1.25	55.0
J. Grensemann^89^	2017	*Eur J Anaesthesiol*	ProAQT	4.0	TPTD	int	critically ill	20	-0.10	1.40	-2.90	2.70	70.0
M. Asamoto^90^ – A	2017	*J Clin Monit Comput*	LiDCOrapid	1.04-b222	PATD	int	mixed	20	-0.45	1.60	-3.65	2.75	53.5
M. Asamoto – B	2017	*J Clin Monit Comput*	FloTrac	3^rd^ Gen (3.02)	PATD	int	mixed	20	-0.38	1.15	-2.69	1.93	74.4
M. Biais^91^	2017	*Anesth Analg*	ProAQT	ns	PATD	cont	liver transplant	30	-0.80	1.65	-4.10	2.50	65.0
M. Lee^92^ – A	2017	*J Clin Monit Comput*	FloTrac	3^rd^ Gen	PATD	int	liver transplant	25	-1.17	1.49	-4.15	1.81	64.4
M. Lee - B	2017	*J Clin Monit Comput*	FloTrac	3^rd^ Gen	PATD	int	liver transplant	25	-0.71	1.51	-3.73	2.31	74.2
GE. Kim^119^	2019	*Yonsei Med J*	FloTrac	ns	PATD	cont	liver transplant	16	-0.62	1.05	-2.71	1.47	93.4
T. Maeda^98^	2019	*J Clin Monit Comput*	FloTrac	4^th^ Gen (4.0)	PATD	int	cardiac surgery	22	0.26	0.54	-0.83	1.35	49.5
B. Khwannimit^100^	2020	*Turk J Med Sci*	FloTrac	4^th^ Gen (4.0)	TPTD	int	critically ill	31	-0.14	0.90	-1.95	1.67	47.4
A. Heijne^104^ – A	2021	*Korean J Anesthesiol*	FloTrac	4^th^ Gen (4.0)	TPTD	int	noncardiac surgery	25	-0.20	0.82	-1.84	1.44	50.0
A. Heijne – B	2021	*Korean J Anesthesiol*	ProAQT	V4.0.0.7	TPTD	int	noncardiac surgery	25	-0.10	0.87	-1.84	1.64	54.0
LA. Ylikauma^108^	2022	*J Cardiothorac Vasc Anesth*	LiDCOrapid	V2.03-318	PATD	int	cardiac surgery	20	0.22	0.59	-0.95	1.39	53.2
LA. Ylikauma^109^	2022	*J Clin Monit Comput*	FloTrac	4^th^ Gen (4.0)	PATD	int	cardiac surgery	20	0.01	0.65	-1.30	1.32	56.8
A. Kee^111^	2023	*J Cardiothorac Vasc Anesth*	ArgosCO	ns	PATD	int	critically ill	22	0.20	0.47	-0.74	1.14	40.0
P. Ordoñez-Rufat^114^	2023	*J Cardiothorac Surg*	ProAQT	ns	PATD	cont	cardiac surgery	10	-0.45	1.05	-2.54	1.64	82.5
Y. Takei^115^ – A	2023	*J Cardiothorac Vasc Anesth*	FloTrac	4^th^ Gen (4.01)	PATD	int	cardiac surgery	29	-0.73	0.72	-2.17	0.71	39.9
Y. Takei – B	2023	*J Cardiothorac Vasc Anesth*	LiDCOrapid	1.04-b251	PATD	int	cardiac surgery	29	-0.61	0.92	-2.46	1.24	51.2
LA. Ylikauma^116^	2023	*BMC Anesthesiol*	LiDCOrapid	ns	PATD	int	noncardiac surgery	23	0.26	0.81	-1.36	1.88	57.1
HPO. Ronkainen^117^	2024	*J Cardiothorac Vasc Anesth*	LiDCOrapid	2.03–318	PATD	int	cardiac surgery	40	-0.15	0.72	-1.59	1.29	68.7
**Summary**								**1188**	**-0.14**	**0.85**	**-1.83**	**1.56**	**49.1**

Seventy-six data sets investigated the FloTrac, 12 the MostCare, 4 the ArgosCO, 10 the LiDCOrapid, and 11 the ProAQT. The firmware version used was not always reported (only in 78/119 data sets), especially for the FloTrac.

### Overall meta-analysis

**Fig 2:**
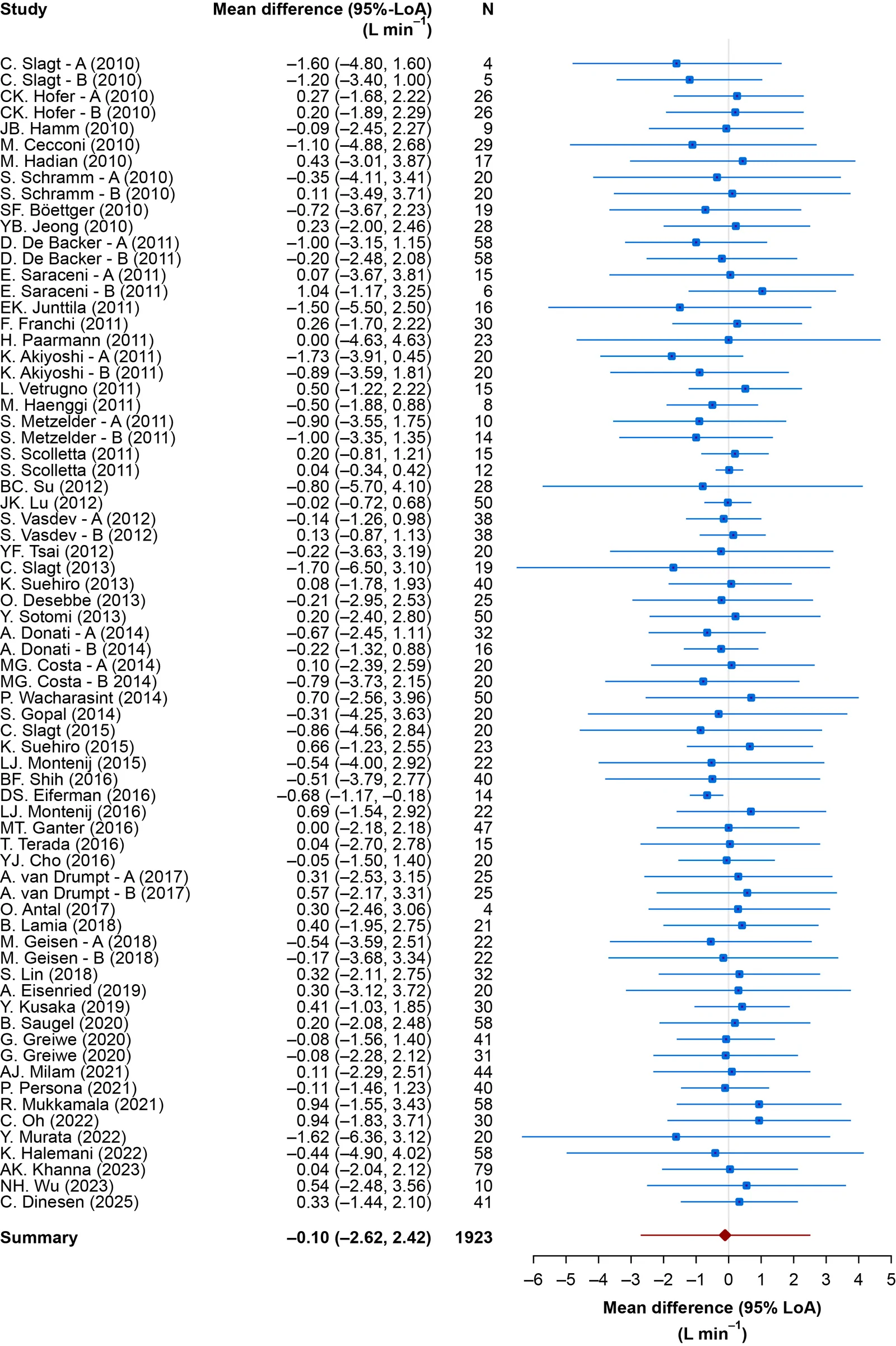
Forest plot showing the results of the meta-analysis for data sets reporting cardiac output. 95% LoA, 95% limits of agreement.

For studies reporting cardiac output, the pooled percentage error (95% CI) was 44.0% (38.2%–49.8%). The pooled mean difference was -0.1 L min^−1^ with a standard deviation of 1.3 L min^−1^ and 95% LoA of -2.6 to 2.4 L min^−1^ (I^2^=9.8%) (Fig. 2, Table 2).

**Table 2:** Summary of the results. Summary of pooled Bland-Altman agreement statistics from the random-effects meta-analysis comparing minimally invasive pulse wave analysisderived cardiac output and cardiac index measurements with reference measurements by pulmonary artery thermodilution or transpulmonary thermodilution, presented overall and stratified by patient population and test device. The number of data sets across subgroups may not add up to the overall total, as meta-analysis was not possible for subgroups with too few data sets.

	Data sets, *n*	Mean difference (Bias)	95% Limits of agreement	Percentage error (95% confidence interval), %
**Cardiac output, L min^−1^**				
Overall	71	-0.10	-2.62, 2.42	44.0 (38.2, 49.8)
*By patient population*				
Cardiac surgery	39	0.19	-2.08, 2.47	47.8 (41.1, 54.6)
Critically ill	22	-0.49	-2.96, 1.98	34.9 (26.1, 43.7)
Liver transplant	9	-0.74	-4.36, 2.87	49.5 (31.6, 67.4)
*By test device*				
FloTrac	54	-0.14	-2.82, 2.55	45.9 (38.9, 52.8)
MostCare	9	-0.08	-1.74, 1.58	30.5 (16.2, 44.9)
ArgosCO	3	0.07	-1.98, 2.12	41.8 (34.4, 49.1)
ProAQT	2	0.45	-2.37, 3.27	47.7 (43.9, 51.6)
**Cardiac index, L min^−1^ m^-2^**				
Overall	42	-0.14	-1.83, 1.56	49.1 (40.6, 57.6)
*By patient population*				
Cardiac surgery	17	-0.05	-1.37, 1.26	46.2 (36.9, 55.5)
Critically ill	13	-0.03	-1.70, 1.63	51.0 (37.2, 64.8)
Liver transplant	5	-1.00	-4.04, 2.04	62.4 (53.7, 71.2)
Noncardiac surgery	5	-0.16	-2.05, 1.74	52.1 (34.1, 70.1)
*By test device*				
FloTrac	22	-0.25	-2.00, 1.50	45.2 (34.8, 55.6)
ProAQT	9	-0.05	-1.89, 1.78	59.7 (34.2, 85.3)
LiDCOrapid	7	-0.03	-1.89, 1.83	63.8 (48.4, 79.3)
MostCare	3	-0.07	-1.08, 0.95	36.6 (17.6, 55.5)

**Fig 3:**
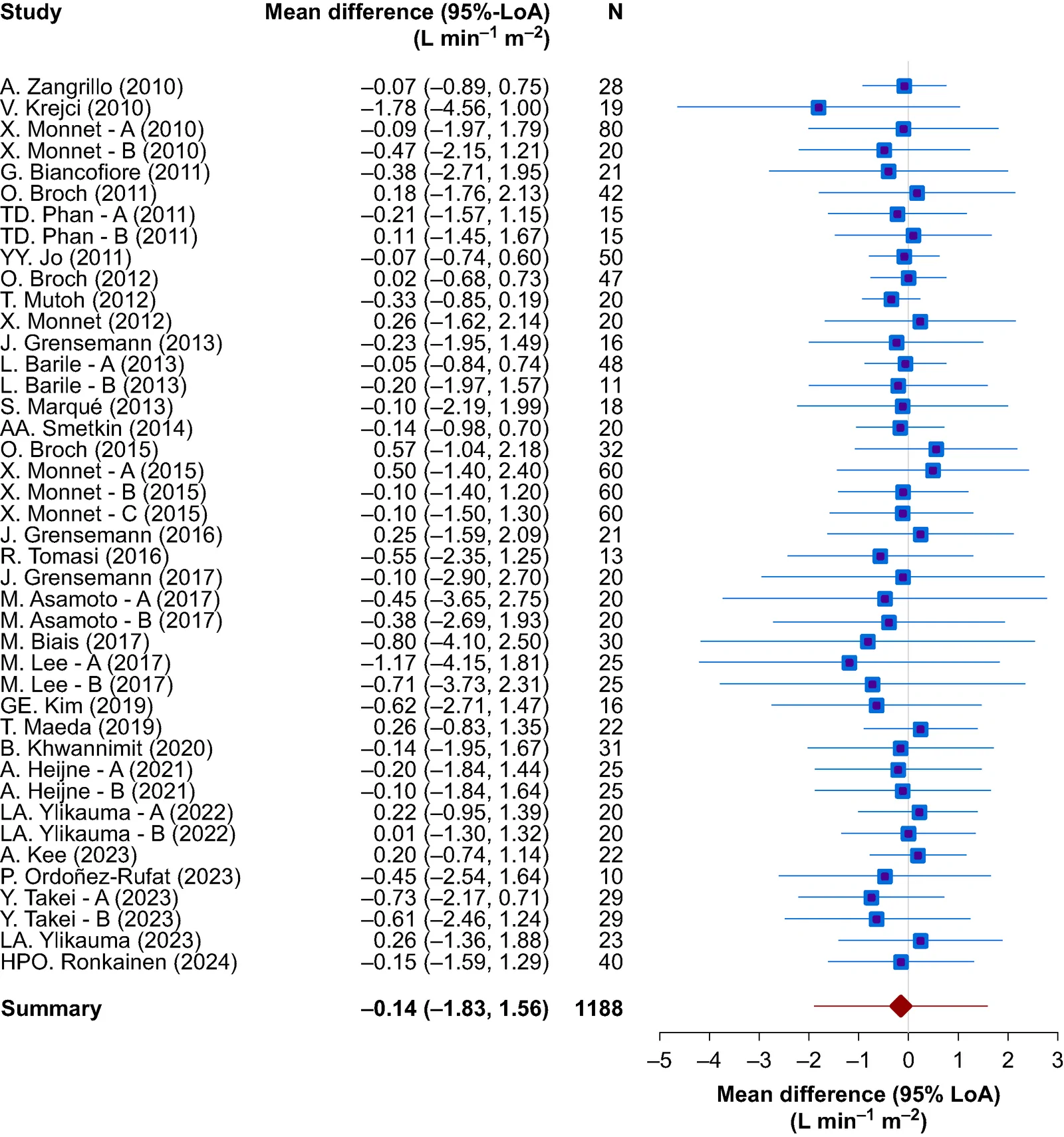
Forest plot showing the results of the meta-analysis for data sets reporting cardiac index measured in L min^−1^ m^−2^. 95% LoA, 95% limits of agreement.

For studies reporting cardiac index, the pooled percentage error was 49.1% (40.6%–57.6%). The pooled mean difference was -0.1 L min^−1^ m^−2^ with a standard deviation of 0.9 L min^−1^ m^−2^ and 95% LoA of -1.8 to 1.6 L min^−1^ m^−2^ (I^2^=7.9%) (Fig. 3, Table 2).

### Subgroup analyses

#### Patient population

For studies reporting cardiac output (Supplementary material S4), the pooled percentage error (95% CI) was 47.8% (41.1%–54.6%; I^2^=4.0%) in cardiac surgery patients (39 data sets), 34.9% (26.1%–43.7%; I^2^=12.4%) in critically ill patients (22 data sets), and 49.5% (31.6%–67.4%; I^2^=9.4%) in liver transplant patients (nine data sets). The number of studies reporting cardiac output in noncardiac surgery patients was insufficient for performing a meta-analysis.

For studies reporting cardiac index (Supplementary material S5), the pooled percentage error was 46.2% (36.9%–55.5%; I^2^=8.4%) in cardiac surgery patients (17 data sets), 51.0% (37.2%–64.8%; I^2^=8.2%) in critically ill patients (13 data sets), 62.4% (53.7%–71.2%; I^2^=5.4%) in liver transplant patients (five data sets), and 52.1% (34.1%–70.1%; I^2^=5.6%) in noncardiac surgery patients (five data sets). The number of studies reporting cardiac index in mixed patient populations was insufficient for performing a meta-analysis.

#### Test device

For studies reporting cardiac output (Supplementary material S6), the pooled percentage error (95% CI) was 45.9% (38.9%–52.8%; I^2^=12.4%) for the FloTrac (54 data sets), 30.5% (16.2%–44.9%; I^2^=5.6%) for the MostCare (nine data sets), 41.8% (34.4%–49.1%; I^2^=0.7%) for the ArgosCO (three data sets), and 47.7% (43.9%–51.6%; I^2^=2.2%) for the ProAQT (two data sets). The number of data sets reporting the percentage error for the LiDCOrapid was insufficient for performing a meta-analysis.

For studies reporting cardiac index (Supplementary material S7), the pooled percentage error was 45.2% (34.8%–55.6%; I^2^=10.5%) for the FloTrac (22 data sets), 36.6% (17.6%–55.5%; I^2^=2.7%) for the MostCare (three data sets), 59.7% (34.2%–85.3%; I^2^=6.5%) for the ProAQT (nine data sets), and 63.8% (48.4%–79.3%; I^2^=8.5%) for the LiDCOrapid (seven data sets). The number of data sets reporting the percentage error for the ArgosCO was insufficient for performing a meta-analysis.

### Risk of bias across studies and sensitivity analysis

The overall risk of bias in the individual studies was low based on the QUADAS-2 tool (Supplementary material S8).

In the sensitivity analysis, we excluded 15 studies in which the risk of bias was rated unclear in at least one domain of the QUADAS-2. The remaining 77 studies were separated into 60 data sets reporting cardiac output with a total of 1635 patients (22 [20 to 31] patients per data set) and 32 data sets reporting cardiac index with a total of 867 patients (22 [20 to 30] patients per data set). In this sensitivity analysis, the pooled percentage error (95% CI) was 46.8% (40.9%–52.8%) for studies reporting cardiac output. The pooled mean difference was -0.1 L min^−1^ with a standard deviation of 1.4 L min^−1^ and a 95% LoA of -2.8 to 2.6 L min^−1^ (I^2^=9.7%). For studies reporting cardiac index, the pooled percentage error was 49.6% (39.9%–59.3%). The pooled mean difference was -0.2 L min^−1^ m^−2^ with a standard deviation of 0.8 L min^−1^ m^−2^ and a 95% LoA of -1.8 to 1.4 L min^−1^ m^−2^ (I^2^=8.2%).

In the additional quality assessment of methods and result reports, only four out of 92 studies fulfilled all assessed domains (Supplementary material S9).

## Discussion

In this systematic review and meta-analysis of studies in adult surgical or critically ill patients, the pooled percentage error between minimally invasive pulse wave analysis-derived cardiac output/cardiac index measurements and reference measurements by pulmonary artery thermodilution or transpulmonary thermodilution was 44.0% for studies reporting cardiac output and 49.1% for studies reporting cardiac index, both exceeding the generally used 30%-threshold for clinically acceptable agreement.^122^ However, the percentage error varied depending on the patient population and device used.

In 2010, a meta-analysis of 24 studies comparing cardiac output measured with minimally invasive pulse wave analysis *vs* thermodilution in surgical and critically ill patients reported a mean weighted percentage error of 41.3%.^19^ To avoid overlap with this previous meta-analysis and to account for advances in device technology and algorithm development, we restricted our analysis to studies published since 2010. Nevertheless, the pooled percentage errors of 44.0% (cardiac output) and 49.1% (cardiac index) we found in our meta-analysis are higher than the mean weighted percentage error of 41.3% in the 2010 meta-analysis.^19^ In contrast to the 2010 meta-analysis that also included pulse wave analysis calibrated to transpulmonary thermodilution,^19^ we only included internally calibrated or uncalibrated pulse wave analysis devices.^13^, ^14^, ^15^ This distinction is important because calibrating pulse wave analysis-derived cardiac output measurements to external transpulmonary thermodilution cardiac output measurements improves the measurement performance – especially when these calibrated values are then compared with transpulmonary thermodilution cardiac output measurements.^123^^,^^124^ Internally calibrated devices often consider demographic and biometric data for cardiac output estimation, whereas uncalibrated devices estimate cardiac output solely based on the arterial pressure waveform.^13^, ^14^, ^15^

The percentage error based on the Bland–Altman analysis is widely accepted as the primary measure to determine clinically acceptable agreement in cardiac output method comparison studies.^21^^,^^26^ What constitutes ‘clinically acceptable agreement’ between cardiac output monitoring methods remains a subject of ongoing scientific debate. Based on the landmark paper by Critchley and Critchley,^122^ a percentage error of 28.3%, often rounded to 30%, is generally used to define clinically acceptable agreement. This percentage error threshold is based on the assumption that both the test and the reference method have a precision of method of 20%. The precision of method reflects to which extent repeated measurements of the same sample with a given method under identical experimental conditions yield consistent results.^125^^,^^126^ In most cases, the precision of method for minimally invasive pulse wave analysis devices is unclear. Therefore, it has been suggested to apply a higher threshold of 45% to define clinically acceptable agreement between minimally invasive pulse wave analysis-derived cardiac output measurements and reference measurements by pulmonary artery thermodilution or transpulmonary thermodilution.^19^ In our meta-analysis, however, the pooled percentage error was slightly outside the 45% threshold. Restricting our analysis to studies with a low risk of bias according to the QUADAS-2 did not substantially alter these results.

Although the percentage error is usually the primary measure to define clinically acceptable agreement in cardiac output method comparison studies, it only reflects the precision of measurements. The percentage error should, therefore, always be interpreted together with the mean difference (or bias) and its 95% LoA. The mean difference in our meta-analysis was low indicating a high accuracy, or trueness, without a systematic measurement error. There was a minor difference between the pooled percentage error for studies reporting cardiac output and studies reporting cardiac index. Considering that indexing cardiac output per body surface area should not affect the percentage error, it seems more likely that this difference was caused by differences in the total number of included studies, patient population, and/or investigated devices.

In our meta-analysis, the percentage error varied depending on the patient population and device used. Most studies were performed in cardiac surgery patients and critically ill patients in whom invasive reference methods are routinely used. However, the haemodynamic profiles of these patients differ substantially from those of patients having noncardiac surgery, in whom minimally invasive pulse wave analysis is commonly used to measure and optimise cardiac output.^16^^,^^127^, ^128^, ^129^ Therefore, a key limitation remains: most cardiac output method comparison studies have not been conducted in the patient population in which these methods are routinely used – namely, patients having major noncardiac surgery.

Consequently, this meta-analysis does not allow recommendations for or against the use of minimally invasive pulse wave analysis in major noncardiac surgery patients. Although the pooled percentage errors exceeded commonly accepted thresholds for clinically acceptable agreement with thermodilution methods, this result does not imply that minimally invasive pulse wave analysis lacks clinical use. Rather, our findings indicate that absolute measurements should be interpreted in a clinical context together with other haemodynamic variables. In addition, pulse wave analysis provides additional haemodynamic variables that may help assess preload dependency (e.g. pulse pressure variation and stroke volume variation), contractility (dP/dt), or afterload (systemic vascular resistance).

The variation in percentage error among the analysed devices reflects differences in the underlying models and algorithms used to estimate cardiac output. This variation is further influenced by the unequal number of studies investigating each device and by differences in patient populations in which they were evaluated. Consequently, the currently available evidence, including the results of our meta-analysis, does not support meaningful direct comparisons between the available devices.

When interpreting the results of this meta-analysis and the included method comparison studies, it is important to recognise conflicts of interest of the authors. Researchers with earlier or ongoing collaborative relationships with manufacturers may, consciously or not, be influenced when designing or performing their studies. At the same time, such relationships can confer legitimate advantages, including earlier access to devices and dedicated technical support during data collection, which may in turn improve the methodological quality and reliability of the research. Conflicts of interest, when adequately declared, should not automatically diminish or invalidate research findings but rather be considered as one factor among many when critically appraising the evidence.

We restricted our meta-analysis to studies that used pulmonary artery thermodilution or transpulmonary thermodilution as the reference method. Both methods are invasive and widely regarded as clinical ‘gold standard’.^9^ This approach minimises heterogeneity between studies and avoids potential bias introduced by alternative reference methods such as Doppler ultrasound, echocardiography, or indirect Fick calculations, which differ in terms of accuracy and reproducibility. Therefore, observed differences between tests and reference cardiac output measurements can be attributed to the test methods themselves rather than to variability in the reference methods. As a result, the observed variation in results among the included studies in this meta-analysis was low, as indicated by a low I^2^. In addition, the quality of the included studies was generally high, and most studies had a low risk of bias and low concern for applicability. However, additional quality assessment found that hardly any studies fulfilled all quality criteria. With a recently published Enhancing the QUAlity and Transparency Of health Research guideline, ‘Statistical Analysis and Reporting of Cardiac Output Method Comparison Studies (COMPARE) Statement’^21^ this will hopefully improve.

Still, this meta-analysis has limitations. Deviating from the initial registration, we performed an additional literature search of the Web of Science and Cochrane databases upon reviewer request. We carefully screened the literature but cannot rule out that we missed eligible studies indexed exclusively in other databases. Also, the random-effects model used to estimate the mean measurement of the reference method ignores the number of repeated measurements and conservatively takes the number of subjects as sample size. As firmware versions for the investigated devices were not always reported, this was not considered for this meta-analysis but may have influenced the measurement performance. Finally, we only investigated the absolute agreement between minimally invasive pulse wave analysis-derived cardiac output or cardiac index measurements and reference measurements by pulmonary artery thermodilution or transpulmonary thermodilution and did not analyse trending. This is relevant, as the trending ability is important when minimally invasive pulse wave analysis is used to assess fluid responsiveness.

In conclusion, the pooled percentage error between minimally invasive pulse wave analysis-derived cardiac output measurements and reference measurements by pulmonary artery thermodilution or transpulmonary thermodilution was 44.0% for studies reporting cardiac output and 49.1% for studies reporting cardiac index in adult surgical or critically ill patients, both exceeding the generally used 30% threshold for clinically acceptable agreement. However, the percentage error varied depending on the patient population and device used. There is a notable lack of studies validating cardiac output measurements by minimally invasive pulse wave analysis in noncardiac surgery patients, the primary target population for this cardiac output monitoring method.

## Authors’ contributions

Study conception/design: MF, BS

Data acquisition: MF, DXM, AB, PS, ME

Data analysis: all authors

Drafting of the manuscript: MF, AH, BS

Critical revision of the manuscript for important intellectual content: all authors.

All authors reviewed the results and approved the final version of the manuscript.

## Declaration of generative AI and AI-assisted technologies in the writing process

During the preparation of this work, the authors used Claude Sonnet 4.5 (Anthropic; San Francisco, CA, USA) to improve readability and language. The authors reviewed and edited the content as needed and take full responsibility for the content of the publication.

## Funding

Institutional sources.

## Declarations of interest

MF is a consultant for Edwards Lifesciences (Irvine, CA, USA) and Vygon (Écouen, France) and has received honoraria for consulting and giving lectures from CNSystems Medizintechnik (Graz, Austria). KK is a consultant for and has received honoraria for giving lectures from Edwards Lifesciences (Irvine, CA, USA). KK is a consultant for Vygon (Aachen, Germany). AB has no conflict of interest to declare. BS is a consultant for Edwards Lifesciences (Irvine, CA, USA), Philips North America (Cambridge, MA, USA), GE Healthcare (Chicago, IL, USA), Vygon (Aachen, Germany), Masimo (Neuchâtel, Switzerland), Retia Medical (Valhalla, NY, USA), Maquet Critical Care (Solna, Sweden), Pulsion Medical Systems (Feldkirchen, Germany), Dynocardia (Cambridge, MA, USA), RDS (Strasbourg, France). BS has received institutional restricted research grants from Edwards Lifesciences, Baxter (Deerfield, IL, USA), GE Healthcare, Masimo, Philips Medizin Systeme Böblingen (Böblingen, Germany), CNSystems Medizintechnik (Graz, Austria), Pulsion Medical Systems, Vygon, Retia Medical, Osypka Medical (Berlin, Germany). BS has received honoraria for giving lectures from Edwards Lifesciences, Philips Medizin Systeme Böblingen, Baxter, GE Healthcare, Masimo, CNSystems Medizintechnik, Getinge (Gothenburg, Sweden), Pulsion Medical Systems, Vygon, Ratiopharm (Ulm, Germany). BS is an editor of the *British Journal of Anaesthesia*. The other authors have no conflicts of interest to declare.
