# Supplementary material for: Agreement of minimally invasive pulse wave analysis with pulmonary artery and transpulmonary thermodilution cardiac output measurements in perioperative and intensive care medicine: a systematic review and meta-analysis
Source: Br J Anaesth. 2026 Jun 2;137(1):39–54. doi: 10.1016/j.bja.2026.04.045 (PMC13494206; doi:10.1016/j.bja.2026.04.045)
Supplement: Supplementary file 1 [file mmc1.pdf]

Search Strategy

Pubmed:

1. ("thermodilution" OR "pulmonary artery catheter" OR "pulse contour analysis" OR "pulse wave analysis" OR "arterial pressure waveform" OR "FloTrac" OR "Vigileo" OR "PiCCO" OR "ProAQT" OR "PulsioFlex" OR "Argos" OR "Retia" OR "MostCare" OR "PRAM" OR “Pressure recording analytical method” OR "LiDCO" OR "LiDCOrapid" OR “Windkessel” OR “Modelflow” OR “Long time interval” OR “pulse power analysis”)
2. AND ("cardiac output" OR "cardiac index" OR "stroke volume" OR "stroke volume index") AND ("Bland-Altman" OR "comparison" OR "performance" OR "evaluation" OR "accuracy" OR "precision" OR "bias" OR "mean difference" OR "mean of the differences" OR "standard deviation" OR "agreement" OR "validation" OR "reproducibility")
3. AND ("2010"[Date - Publication] : "2025"[Date - Publication])
4. NOT ("neonates" OR "neonate" OR "neonatal" OR "newborn" OR "newborns" OR "infants" OR "infant" OR "child" OR "children" OR "pediatric" OR "pediatrics" OR "paediatric" OR "paediatrics" OR "animals" OR "animal" OR "sheep" OR "sheeps" OR "horse" OR "horses" OR "dog" OR "dogs" OR "cat" OR "cats" OR "pig" OR "pigs" OR "swine" OR "swines")
5. NOT (review[Publication Type])

Web of Science:

ALL=("thermodilution" OR "pulmonary artery catheter" OR "pulse contour analysis" OR "pulse wave analysis" OR "arterial pressure waveform" OR "FloTrac" OR "Vigileo" OR "PiCCO" OR "ProAQT" OR "PulsioFlex" OR "Argos" OR "Retia" OR "MostCare" OR "PRAM" OR

"Pressure recording analytical method" OR "LiDCO" OR "LiDCOrapid" OR "Windkessel" OR

"Modelflow" OR "Long time interval" OR "pulse power analysis")

AND

ALL=("cardiac output" OR "cardiac index" OR "stroke volume" OR "stroke volume index")

AND

ALL=("Bland-Altman" OR "comparison" OR "performance" OR "evaluation" OR "accuracy" OR

"precision" OR "bias" OR "mean difference" OR "mean of the differences" OR

"standard deviation" OR "agreement" OR "validation" OR "reproducibility")

NOT

ALL=("neonates" OR "neonate" OR "neonatal" OR "newborn" OR "newborns" OR "infants" OR

"infant" OR "child" OR "children" OR "pediatric" OR "pediatrics" OR "paediatric" OR

"paediatrics" OR "animals" OR "animal" OR "sheep" OR "sheeps" OR "horse" OR "horses"

OR "dog" OR "dogs" OR "cat" OR "cats" OR "pig" OR "pigs" OR "swine" OR "swines")

NOT DT=(Review)

Cochrane

1. ("thermodilution" OR "pulmonary artery catheter" OR "pulse contour analysis" OR "pulse wave analysis" OR "arterial pressure waveform" OR "FloTrac" OR "Vigileo" OR "PiCCO" OR "ProAQT" OR "PulsioFlex" OR "Argos" OR "Retia" OR "MostCare" OR "PRAM" OR “Pressure recording analytical method” OR "LiDCO" OR "LiDCOrapid" OR “Windkessel” OR “Modelflow” OR “Long time interval” OR “pulse power analysis”)
2. AND ("cardiac output" OR "cardiac index" OR "stroke volume" OR "stroke volume index") AND ("Bland-Altman" OR "comparison" OR "performance" OR "evaluation" OR "accuracy" OR "precision" OR "bias" OR "mean difference" OR "mean of the differences" OR "standard deviation" OR "agreement" OR "validation" OR "reproducibility")
3. NOT ("neonates" OR "neonate" OR "neonatal" OR "newborn" OR "newborns" OR "infants" OR "infant" OR "child" OR "children" OR "pediatric" OR "pediatrics" OR "paediatric" OR "paediatrics" OR "animals" OR "animal" OR "sheep" OR "sheeps" OR "horse" OR "horses" OR "dog" OR "dogs" OR "cat" OR "cats" OR "pig" OR "pigs" OR "swine" OR "swines")
4. NOT (review[Publication Type])

with Cochrane Library publication date from Jan 2010 to Jul 2025, (Word variations have been searched)
