# Supplementary material for: Agreement of minimally invasive pulse wave analysis with pulmonary artery and transpulmonary thermodilution cardiac output measurements in perioperative and intensive care medicine: a systematic review and meta-analysis
Source: Br J Anaesth. 2026 Jun 2;137(1):39–54. doi: 10.1016/j.bja.2026.04.045 (PMC13494206; doi:10.1016/j.bja.2026.04.045)
Supplement: Supplementary file 2 [file mmc2.pdf]

Risk of bias is judged as “Low,” “High,” or “Unclear.” If the answers to all signaling questions for a domain are “Yes,” then risk of bias can be judged low. If any signaling question is answered “no,” potential for bias exists. Review authors must then use the guidelines developed in phase 2 to judge risk of bias. The “unclear” category should be used only when insufficient data are reported to permit a judgment.

Risk of Bias

**1) Patient selection**			
**Risk of Bias: Could the selection of patients have introduced bias? (Two No = High)**	**Low**	**High**	**Unclear**
Signaling question 1: Did the study describe population of interest and demographic data?	Yes	No	Unclear
Signaling question 2: Were the inclusion and exclusion criteria clearly described?	Yes	No	Unclear
**2) Index test (minimally invasive pulse wave analysis cardiac output monitoring)**			
**Could the conduct or interpretation of pulse wave analysis cardiac output/index (CO/CI) monitoring have introduced bias? (No = High)**	**Low**	**High**	**Unclear**
Signaling question 1: Was the setting of pulse wave analysis CO/CI monitoring described clearly? (for example, position, calibration, manufacturer, characteristics etc.)	Yes	No	Unclear
**3) Reference standard (transpulmonary/pulmonary thermodilution for CO/CI)**			
**Could the reference method, its conduct, or its interpretation have introduced bias? (No = High)**	**Low**	**High**	**Unclear**
Signaling question 1 CO/CI: Is the reference CO/CI monitoring likely to correctly measure CO/CI?	Yes	No	Unclear
**4) Flow and timing**			
**Could the patient flow have introduced bias? (Could the analysis of flow and timing have introduced bias? (Unclear: ≥2 unclear, 1 No + 1 unclear) (High: ≥2 No)**	**Low**	**High**	**Unclear**
Signaling question 1: Were the number of patients enrolled and who dropped out clearly described in the results?	Yes	No	Unclear
Signaling question 2: Were the reference and index test measured simultaneously?	Yes	No	Unclear
Signaling question 3: Were the methods of acquiring paired measurements well described?	Yes	No	Unclear
Signaling question 4: In case of repeated measurements of CO/CI in patients, did they use statistical analysis for agreement between methods of measurement with multiple observations per individual?	Yes	No	Unclear

Applicability

1) **Patient selection**			
Are there concerns that the included patients and setting do not match the review question?	Low	High	Unclear
2) **Index test (minimally invasive pulse wave analysis cardiac output monitoring)**			
Are there concerns that the index tests, their conduct, or interpretation differ from the review question?	Low	High	Unclear
3) **Reference standard (transpulmonary/pulmonary thermodilution for CO/CI)**			
Is there concern that the target condition as defined by the reference standard does not match the review question?	Low	High	Unclear

References

1. Saugel B, Hoppe P, Nicklas JY, et al. Continuous noninvasive pulse wave analysis using finger cuff technologies for arterial blood pressure and cardiac output monitoring in perioperative and intensive care medicine: a systematic review and meta-analysis. Br J Anaesth 2020; 125: 25-37

2. Suehiro K, Joosten A, Murphy LS, et al. Accuracy and precision of minimally-invasive cardiac output monitoring in children: a systematic review and meta-analysis. J Clin Monit Comput 2016; 30: 603-20

3. Whiting PF, Rutjes AW, Westwood ME, et al. QUADAS-2: a revised tool for the quality assessment of diagnostic accuracy studies. Ann Intern Med 2011; 155: 529-36
