# Supplementary material for: Agreement of minimally invasive pulse wave analysis with pulmonary artery and transpulmonary thermodilution cardiac output measurements in perioperative and intensive care medicine: a systematic review and meta-analysis
Source: Br J Anaesth. 2026 Jun 2;137(1):39–54. doi: 10.1016/j.bja.2026.04.045 (PMC13494206; doi:10.1016/j.bja.2026.04.045)
Supplement: Supplementary file 3 [file mmc3.pdf]

List of excluded articles after full text screening

Externally calibrated pulse wave analysis only (n=5):

1. Fischer, M.O., et al., Assessment of changes in cardiac index with calibrated pulse contour analysis in cardiac surgery: A prospective observational study. Anaesth Crit Care Pain Med, 2016. 35(4): p. 261-7.

2. Menger, J., et al., Accuracy of Continuous Cardiac Output Measurement With the LiDCOplus System During Intra-Aortic Counterpulsation After Cardiac Surgery. J Cardiothorac Vasc Anesth, 2016. 30(3): p. 592-8.

3. Park, M., et al., Evaluation of New Calibrated Pulse-Wave Analysis (VolumeViewTM/EV1000TM) for Cardiac Output Monitoring Undergoing Living Donor Liver Transplantation. PLoS One, 2016. 11(10): p. e0164521.

4. Vetrugno, L., et al., Cardiac output measurement in liver transplantation patients using pulmonary and transpulmonary thermodilution: a comparative study. J Clin Monit Comput, 2019. 33(2): p. 223-231.

5. Gruenewald, M., et al., Effect of norepinephrine dosage and calibration frequency on accuracy of pulse contour-derived cardiac output. Crit Care, 2011. 15(1): p. R22.

No test device (n=2):

6. Yang, H.L., et al., Development and Validation of an Arterial Pressure-Based Cardiac Output Algorithm Using a Convolutional Neural Network: Retrospective Study Based on Prospective Registry Data. JMIR Med Inform, 2021. 9(8): p. e24762.

7. Broch, O., et al., Accuracy of Cardiac Output by Nine Different Pulse Contour Algorithms in Cardiac Surgery Patients: A Comparison with Transpulmonary Thermodilution. Biomed Res Int, 2016. 2016: p. 3468015.

Insufficient hemodynamic data (n=3):

8. Tejedor, A., et al., Accuracy of Vigileo/Flotrac monitoring system in morbidly obese patients. J Crit Care, 2015. 30(3): p. 562-6.

9. Suehiro, K., et al., The ability of the Vigileo-FloTrac system to measure cardiac output and track cardiac output changes during one-lung ventilation. J Clin Monit Comput, 2015. 29(3): p. 333-9.

10. Petzoldt, M., et al., Stroke volume determination using transcardiopulmonary thermodilution and arterial pulse contour analysis in severe aortic valve disease. Intensive Care Med, 2013. 39(4): p. 601-11.

Article not in English (n=1)

11. Ünver, S. et al., Comparison of cardiac output measurements obtained through thermo dilution method and arterial pressure wave analysis in patients undergoing cardiopulmonary bypass graft surgery. Turk J Thorac Cardiovasc Surg, 2012. 20(1): p. 32-8
