# Supplementary material for: Agreement of minimally invasive pulse wave analysis with pulmonary artery and transpulmonary thermodilution cardiac output measurements in perioperative and intensive care medicine: a systematic review and meta-analysis
Source: Br J Anaesth. 2026 Jun 2;137(1):39–54. doi: 10.1016/j.bja.2026.04.045 (PMC13494206; doi:10.1016/j.bja.2026.04.045)
Supplement: Supplementary file 4 [file mmc4.pdf]

Supplement S4. Forest plot showing subgroup analysis for studies reporting cardiac output by patient population

Population	Data sets	Mean difference (95% LoA) (L/min)	Percentage error (%)
Cardiac surgery	39	0.19 (−2.08, 2.47)	47.8 (41.1, 54.6)
Critically ill	22	−0.49 (−2.96, 1.98)	34.9 (26.1, 43.7)
Liver transplant	9	−0.74 (−4.36, 2.87)	49.5 (31.6, 67.4)

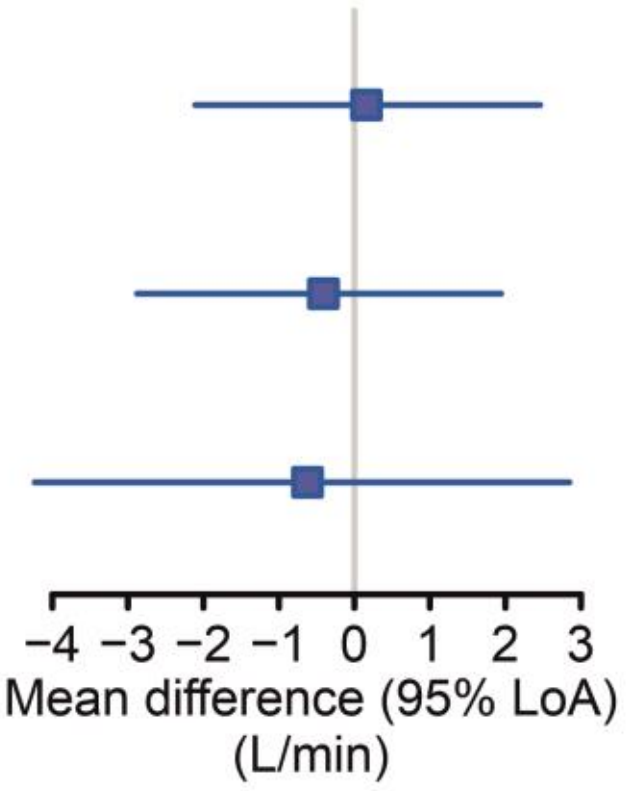

95 LoA – 95%-Limits of Agreement
