# Supplementary material for: Agreement of minimally invasive pulse wave analysis with pulmonary artery and transpulmonary thermodilution cardiac output measurements in perioperative and intensive care medicine: a systematic review and meta-analysis
Source: Br J Anaesth. 2026 Jun 2;137(1):39–54. doi: 10.1016/j.bja.2026.04.045 (PMC13494206; doi:10.1016/j.bja.2026.04.045)
Supplement: Supplementary file 5 [file mmc5.pdf]

Supplement S5. Forest plot showing subgroup analysis for studies reporting cardiac index by patient population

Population	Data sets	Mean difference (95% LoA) (L/min/m²)	Percentage error (%)
Cardiac surgery	17	−0.05 (−1.37, 1.26)	46.2 (36.9, 55.5)
Critically ill	13	−0.03 (−1.70, 1.63)	51.0 (37.2, 64.8)
Liver transplant	5	−1.00 (−4.04, 2.04)	62.4 (53.7, 71.2)
Non−cardiac surgery	5	−0.16 (−2.05, 1.74)	52.1 (34.1, 70.1)

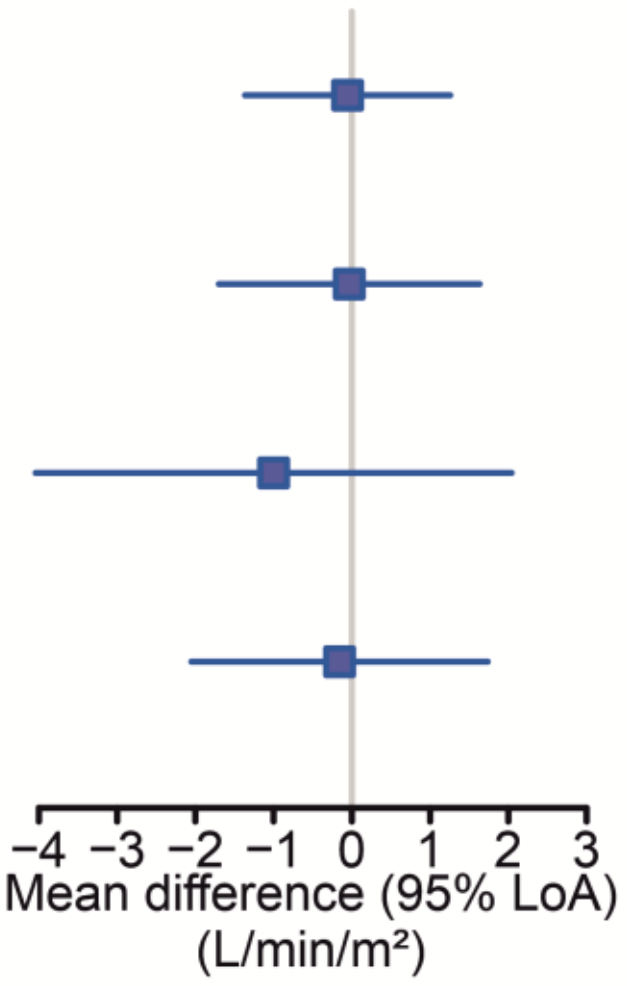

95 LoA – 95%-Limits of Agreement
