# Supplementary material for: Agreement of minimally invasive pulse wave analysis with pulmonary artery and transpulmonary thermodilution cardiac output measurements in perioperative and intensive care medicine: a systematic review and meta-analysis
Source: Br J Anaesth. 2026 Jun 2;137(1):39–54. doi: 10.1016/j.bja.2026.04.045 (PMC13494206; doi:10.1016/j.bja.2026.04.045)
Supplement: Supplementary file 6 [file mmc6.pdf]

Supplement S6. Forest plot showing subgroup analysis for studies reporting cardiac output by device

Device	Data sets	Mean difference (95% LoA) (L/min)	Percentage error (%)
FloTrac	54	−0.14 (−2.82, 2.55)	45.9 (38.9, 52.8)
MostCare	9	−0.08 (−1.74, 1.58)	30.5 (16.2, 44.9)
Argos CO	3	0.07 (−1.98, 2.12)	41.8 (34.4, 49.1)
ProAQT	2	0.45 (−2.37, 3.27)	47.7 (43.9, 51.6)

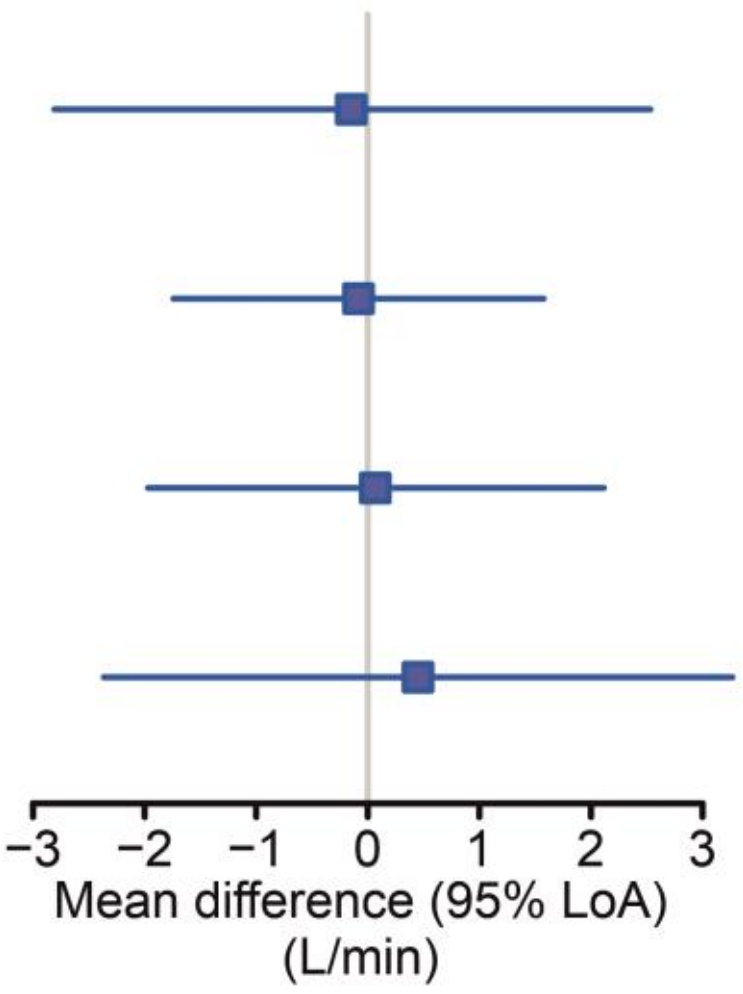

95 LoA – 95%-Limits of Agreement
