# Supplementary material for: Agreement of minimally invasive pulse wave analysis with pulmonary artery and transpulmonary thermodilution cardiac output measurements in perioperative and intensive care medicine: a systematic review and meta-analysis
Source: Br J Anaesth. 2026 Jun 2;137(1):39–54. doi: 10.1016/j.bja.2026.04.045 (PMC13494206; doi:10.1016/j.bja.2026.04.045)
Supplement: Supplementary file 7 [file mmc7.pdf]

Supplement S7. Forest plot showing subgroup analysis for studies reporting cardiac index by device

Device	Data sets	Mean difference (95% LoA) (L/min/m²)	Percentage error (%)
FloTrac	22	−0.25 (−2.00, 1.50)	45.2 (34.8, 55.6)
ProAQT	9	−0.05 (−1.89, 1.78)	59.7 (34.2, 85.3)
LidCOrapid	7	−0.03 (−1.89, 1.83)	63.8 (48.4, 79.3)
MostCare	3	−0.07 (−1.08, 0.95)	36.6 (17.6, 55.5)

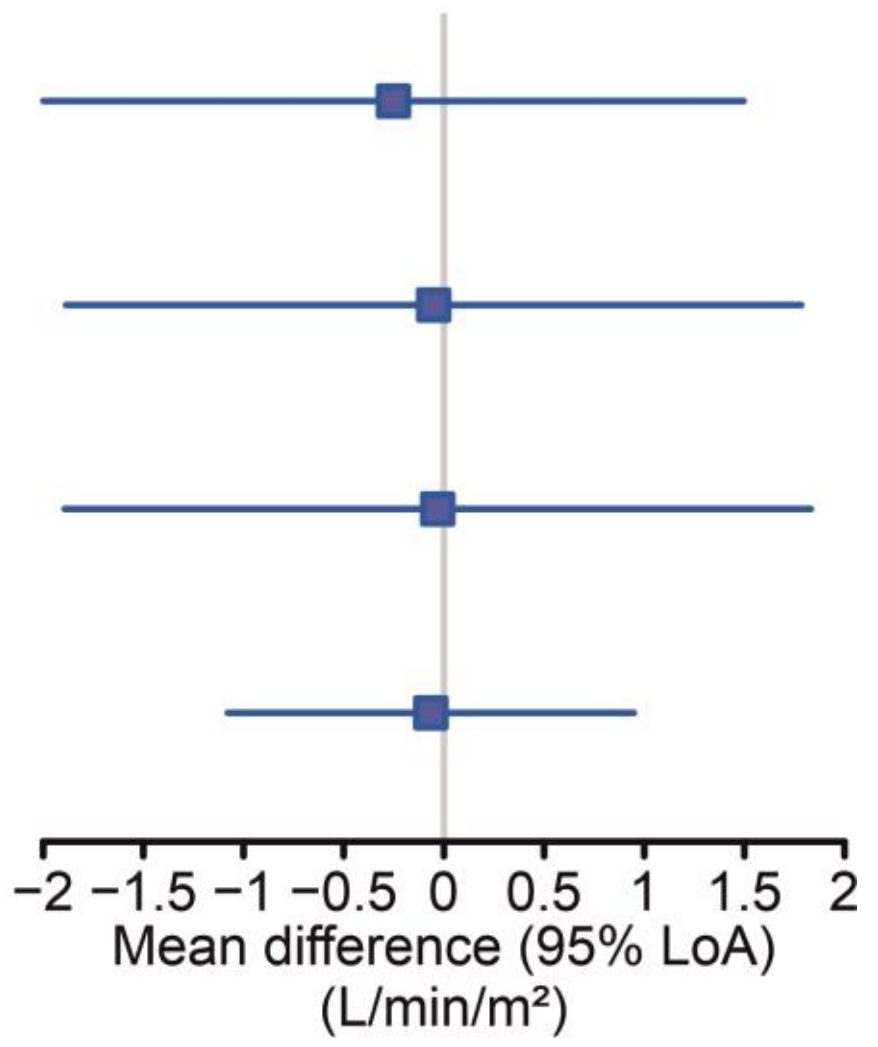

95 LoA – 95%-Limits of Agreement
