# Supplementary material for: Agreement of minimally invasive pulse wave analysis with pulmonary artery and transpulmonary thermodilution cardiac output measurements in perioperative and intensive care medicine: a systematic review and meta-analysis
Source: Br J Anaesth. 2026 Jun 2;137(1):39–54. doi: 10.1016/j.bja.2026.04.045 (PMC13494206; doi:10.1016/j.bja.2026.04.045)
Supplement: Supplementary file 8 [file mmc8.pdf]

Supplement 8 – Risk of Bias assessment

		Risk of Bias				Applicability Concerns		
**Study**	**year**	**#1**	**#2**	**#3**	**#4**	**#5**	**#6**	**#7**
Zangrillo et al.	2010	🙂	🙂	🙂	🙂	🙂	🙂	🙂
Slagt et al.	2010	🙂	🙂	🙂	🙂	🙂	🙂	🙂
Hofer et al.	2010	🙂	🙂	🙂	🙂	🙂	🙂	🙂
Hamm et al.	2010	🙂	🙂	🙂	🙂	🙂	🙂	🙂
Cecconi et al.	2010	🙂	🙂	🙂	🙂	🙂	🙂	🙂
Hadian et al.	2010	🙂	🙂	🙂	🙂	🙂	🙂	🙂
Schramm et al.	2010	🙂	🙂	🙂	🙂	🙂	🙂	🙂
Böettger et al.	2010	🙂	🙂	🙂	🙂	🙂	🙂	🙂
Krejci et al.	2010	🙂	🙂	🙂	🙂	🙂	🙂	🙂
Monnet et al.	2010	🙂	🙂	🙂	🙂	🙂	🙂	🙂
Jeong et al.	2010	🙂	🙂	🙂	🙂	🙂	🙂	🙂
De Backer et al.	2011	🙂	🙂	🙂	🙂	🙂	🙂	🙂
Saraceni et al.	2011	🙂	🙂	🙂	🙂	🙂	🙂	🙂
Junttila et al.	2011	🙂	🙂	🙂	🙂	🙂	🙂	🙂
Franchi et al.	2011	🙂	🙂	🙂	🙂	🙂	🙂	🙂
Biancofiore et al.	2011	🙂	🙂	🙂	🙂	🙂	🙂	🙂
Paarmann et al.	2011	🙂	🙂	🙂	😐	🙂	🙂	🙂
Akiyoshi et al.	2011	🙂	🙂	🙂	🙂	🙂	🙂	🙂
Vetrugno et al.	2011	🙂	🙂	🙂	🙂	🙂	🙂	🙂
Haenggi et al.	2011	🙂	😐	🙂	🙂	🙂	🙂	🙂
Broch et al.	2011	🙂	🙂	🙂	🙂	🙂	🙂	🙂

		Risk of Bias				Applicability Concerns		
**Study**	**year**	**#1**	**#2**	**#3**	**#4**	**#5**	**#6**	**#7**
Metzelder et al.	2011	😊	😊	😊	😊	😊	😊	😊
Scolletta et al.	2011	😊	😊	😊	😊	😊	😊	😊
Scolletta et al.	2011	😊	😊	😊	😊	😊	😊	😊
Phan et al.	2011	😊	😊	😊	😐	😊	😊	😊
Jo et al.	2011	😊	😊	😊	😊	😊	😊	😊
Su et al.	2012	😊	😊	😊	😊	😊	😊	😊
Lu et al.	2012	😊	😊	😊	😐	😊	😊	😊
Broch et al.	2012	😊	😊	😊	😊	😊	😊	😊
Vasdev et al.	2012	😊	😊	😊	😐	😊	😊	😊
Mutoh et al.	2012	😊	😊	😊	😊	😊	😊	😊
Monnet et al.	2012	😊	😊	😊	😐	😊	😊	😊
Tsai et al.	2012	😊	😊	😊	😊	😊	😊	😊
Slagt et al.	2013	😊	😊	😊	😊	😊	😊	😊
Grensemann et al.	2013	😊	😊	😊	😊	😊	😊	😊
Suehiro et al.	2013	😊	😊	😊	😊	😊	😊	😊
Baril et al.	2013	😊	😊	😊	😊	😊	😊	😊
Desebbe et al.	2013	😊	😊	😊	😊	😊	😊	😊
Marqué et al.	2013	😊	😊	😊	😊	😊	😊	😊
Sotomi et al.	2013	😊	😊	😊	😊	😊	😊	😊
Donati et al.	2014	😊	😊	😊	😐	😊	😊	😊
Smetkin et al.	2014	😊	😊	😊	😊	😊	😊	😊
Costa et al.	2014	😊	😊	😊	😊	😊	😊	😊

		Risk of Bias				Applicability Concerns		
**Study**	**year**	**#1**	**#2**	**#3**	**#4**	**#5**	**#6**	**#7**
Wacharasint et al.	2014	🙂	🙂	🙂	😐	🙂	🙂	🙂
Gopal et al.	2014	🙂	🙂	🙂	🙂	🙂	🙂	🙂
Slagt et al.	2015	🙂	🙂	🙂	🙂	🙂	🙂	🙂
Suehiro et al.	2015	🙂	🙂	🙂	🙂	🙂	🙂	🙂
Montenij et al.	2015	🙂	🙂	🙂	🙂	🙂	🙂	🙂
Broch et al.	2015	🙂	🙂	🙂	🙂	🙂	🙂	🙂
Monnet et al.	2015	🙂	🙂	🙂	😐	🙂	🙂	🙂
Shih et al.	2016	🙂	🙂	🙂	🙂	🙂	🙂	🙂
Eiferman et al.	2016	🙂	😐	🙂	😐	🙂	🙂	🙂
Grensemann et al.	2016	🙂	🙂	🙂	😐	🙂	🙂	🙂
Montenij et al	2016	🙂	🙂	🙂	🙂	🙂	🙂	🙂
Ganter et al	2016	🙂	🙂	🙂	🙂	🙂	🙂	🙂
Tomasi et al.	2016	🙂	🙂	🙂	🙂	🙂	🙂	🙂
Terada et al.	2016	🙂	🙂	🙂	😐	🙂	🙂	🙂
Cho et al.	2016	🙂	🙂	🙂	🙂	🙂	🙂	🙂
van Drumpt et al.	2017	🙂	🙂	🙂	🙂	🙂	🙂	🙂
Grensemann et al	2017	😐	🙂	🙂	🙂	🙂	🙂	🙂
Asamoto et al	2017	🙂	🙂	🙂	🙂	🙂	🙂	🙂
Biais et al	2017	🙂	🙂	🙂	🙂	🙂	🙂	🙂
Lee et al.	2017	🙂	🙂	🙂	😐	🙂	🙂	🙂
Antal et al.	2017	🙂	🙂	🙂	😐	🙂	🙂	🙂
Lamia et al.	2018	🙂	🙂	🙂	🙂	🙂	🙂	🙂

		Risk of Bias				Applicability Concerns		
**Study**	**year**	**#1**	**#2**	**#3**	**#4**	**#5**	**#6**	**#7**
Lin et al.	2018	☺	☺	☺	☺	☺	☺	☺
Geisen et al.	2018	☺	☺	☺	☺	☺	☺	☺
Eisenried et al.	2019	☺	☺	☺	☺	☺	☺	☺
Kim et al.	2019	☺	☺	☺	☺	☺	☺	☺
Maeda et al.	2019	☺	☺	☺	☺	☺	☺	☺
Kusaka et al.	2019	☺	☺	☺	☺	☺	☺	☺
Khwannimit et al.	2020	☺	☺	☺	☺	☺	☺	☺
Saugel et al.	2020	☺	☺	☺	☺	☺	☺	☺
Greiwe et al.	2020	☺	☺	☺	☺	☺	☺	☺
Greiwe et al.	2020	☺	☺	☺	☺	☺	☺	☺
Heijne et al.	2021	☺	☺	☺	☺	☺	☺	☺
Milam et al.	2021	☺	☺	☺	☺	☺	☺	☺
Persona et al.	2021	☺	☺	☺	☺	☺	☺	☺
Mukkamala et al.	2021	☺	☺	☺	☺	☺	☺	☺
Oh et al.	2022	☺	☺	☺	☺	☺	☺	☺
Ylikauma et al.	2022	☺	☺	☺	☺	☺	☺	☺
Ylikauma et al.	2022	☺	☺	☺	☺	☺	☺	☺
Halemani et al.	2022	☺	☺	☺	☺	☺	☺	☺
Murata et al.	2022	☺	☺	☺	☺	☺	☺	☺
Kee et al.	2023	☺	☺	☺	☺	☺	☺	☺
Khanna et al.	2023	☺	☺	☺	☺	☺	☺	☺
Wu et al.	2023	☺	☺	☺	☺	☺	☺	☺

		Risk of Bias				Applicability Concerns		
**Study**	**year**	**#1**	**#2**	**#3**	**#4**	**#5**	**#6**	**#7**
Ordoñez-Rufat et al.	2023	🙂	🙂	🙂	🙂	🙂	🙂	🙂
Takei et al.	2023	🙂	🙂	🙂	🙂	🙂	🙂	🙂
Ylikauma et al.	2023	🙂	🙂	🙂	🙂	🙂	🙂	🙂
Ronkainen et al.	2024	🙂	🙂	🙂	🙂	🙂	🙂	🙂
Dinesen et al.	2025	🙂	🙂	🙂	🙂	🙂	🙂	🙂

🙂 = low

😐 = unclear
