# Supplementary material for: Agreement of minimally invasive pulse wave analysis with pulmonary artery and transpulmonary thermodilution cardiac output measurements in perioperative and intensive care medicine: a systematic review and meta-analysis
Source: Br J Anaesth. 2026 Jun 2;137(1):39–54. doi: 10.1016/j.bja.2026.04.045 (PMC13494206; doi:10.1016/j.bja.2026.04.045)
Supplement: Supplementary file 9 [file mmc9.pdf]

Supplement 9 – Quality checklist to assess methods and results

Study	Year	Measurement protocol described	Appropriate reference method	Predefined acceptable error (e.g., 30%)	Sample size justification	Normality check	Bland-Altman analysis	Mean difference + 95%-limits of agreement	Percentage error	Confidence intervals reported	Corrected for repeated measurements	Proportional bias assessment
Zangrillo et al.	2010	Yes	Yes	Yes	No	Yes	Yes	Yes	Yes	No	Not applicable	Yes
Slagt et al.	2010	Yes	Yes	No	No	Yes	Yes	Yes	Yes	No	Yes	No
Hofer et al.	2010	Yes	Yes	No	Yes	Yes	Yes	Yes	No	No	Yes	No
Hamm et al.	2010	Yes	Yes	Yes	No	No	Yes	Yes	Yes	No	No	No
Cecconi et al.	2010	Yes	Yes	No	No	Yes	Yes	Yes	Yes	No	No	No
Hadian et al.	2010	Yes	Yes	No	No	No	Yes	Yes	Yes	No	No	Yes
Schramm et al.	2010	Yes	Yes	Yes	Yes	No	Yes	Yes	Yes	No	No	No
Böettger et al.	2010	Yes	Yes	Yes	No	No	Yes	Yes	Yes	No	Yes	Yes
Krejci et al.	2010	Yes	Yes	No	No	No	Yes	Yes	Yes	Yes	No	Yes
Monnet et al.	2010	Yes	Yes	No	No	Yes	Yes	Yes	Yes	No	Not applicable	Yes
Jeong et al.	2010	Yes	Yes	Yes	No	No	Yes	Yes	Yes	No	Yes	Yes
De Backer et al.	2011	Yes	Yes	Yes	No	No	Yes	Yes	Yes	Yes	Yes	No
Saraceni et al.	2011	Yes	Yes	No	No	No	Yes	Yes	No	No	No	Yes

Study	Year	Measurement protocol described	Appropriate reference method	Predefined acceptable error (e.g., 30%)	Sample size justification	Normality check	Bland-Altman analysis	Mean difference + 95%-limits of agreement	Percentage error	Confidence intervals reported	Corrected for repeated measurements	Proportional bias assessment
Junttila et al.	2011	Yes	Yes	No	No	No	Yes	Yes	Yes	No	Yes	Yes
Franchi et al.	2011	Yes	Yes	No	No	No	Yes	Yes	Yes	No	Yes	Yes
Biancofiore et al.	2011	Yes	Yes	No	No	No	Yes	Yes	Yes	Yes	Yes	Yes
Paarmann et al.	2011	Yes	Yes	Yes	Yes	Yes	Yes	Yes	Yes	Yes	Yes	Yes
Akiyoshi et al.	2011	Yes	Yes	Yes	No	No	Yes	Yes	Yes	No	Yes	No
Vetrugno et al.	2011	Yes	Yes	No	No	No	Yes	Yes	Yes	No	Yes	No
Haenggi et al.	2011	Yes	Yes	Yes	No	No	Yes	Yes	Yes	No	Yes	Yes
Broch et al.	2011	Yes	Yes	No	Yes	No	Yes	Yes	Yes	Yes	No	Yes
Metzelder et al.	2011	Yes	Yes	No	No	yes	Yes	Yes	Yes	No	Yes	Yes
Scolletta et al.	2011	Yes	Yes	No	No	No	Yes	Yes	Yes	Yes	Yes	Yes
Scolletta et al.	2011	Yes	Yes	No	No	No	Yes	Yes	Yes	No	No	Yes
Phan et al.	2011	Yes	Yes	No	No	No	Yes	Yes	Yes	No	Yes	Yes
Jo et al.	2011	Yes	Yes	Yes	No	yes	Yes	Yes	Yes	No	Yes	No
Su et al.	2012	Yes	Yes	Yes	No	No	Yes	Yes	Yes	No	Yes	No

Study	Year	Measurement protocol described	Appropriate reference method	Predefined acceptable error (e.g., 30%)	Sample size justification	Normality check	Bland-Altman analysis	Mean difference + 95%-limits of agreement	Percentage error	Confidence intervals reported	Corrected for repeated measurements	Proportional bias assessment
Lu et al.	2012	Yes	Yes	No	No	No	Yes	Yes	No	No	No	No
Broch et al.	2012	Yes	Yes	No	Yes	No	Yes	Yes	Yes	No	Yes	Yes
Vasdev et al.	2012	Yes	Yes	No	No	No	Yes	Yes	Yes	No	No	No
Mutoh et al.	2012	Yes	Yes	No	Yes	Yes	Yes	No	Yes	No	No	Yes
Monnet et al.	2012	Yes	Yes	No	No	Yes	Yes	Yes	Yes	No	No	Yes
Tsai et al.	2012	Yes	Yes	Yes	No	No	Yes	Yes	Yes	No	Yes	No
Slagt et al.	2013	Yes	Yes	No	Yes	Yes	Yes	Yes	Yes	No	Yes	Yes
Grensemann et al.	2013	Yes	Yes	No	Yes	Yes	Yes	Yes	Yes	No	Yes	Yes
Suehiro et al.	2013	Yes	Yes	Yes	No	No	Yes	Yes	Yes	No	Yes	Yes
Baril et al.	2013	Yes	Yes	No	No	Yes	Yes	Yes	Yes	No	Yes	No
Desebbe et al.	2013	Yes	Yes	Yes	No	No	Yes	Yes	Yes	No	Yes	Yes
Marqué et al.	2013	Yes	Yes	Yes	No	Yes	Yes	Yes	Yes	No	Yes	Yes
Sotomi et al.	2013	Yes	Yes	Yes	No	No	Yes	Yes	Yes	No	Yes	Yes
Donati et al.	2014	Yes	Yes	Yes	No	No	Yes	Yes	Yes	No	No	No

Study	Year	Measurement protocol described	Appropriate reference method	Predefined acceptable error (e.g., 30%)	Sample size justification	Normality check	Bland-Altman analysis	Mean difference + 95%-limits of agreement	Percentage error	Confidence intervals reported	Corrected for repeated measurements	Proportional bias assessment
Smetkin et al.	2014	Yes	Yes	Yes	No	Yes	Yes	Yes	Yes	No	Yes	Yes
Costa et al.	2014	Yes	Yes	Yes	Yes	Yes	Yes	Yes	Yes	No	Yes	No
Wacharasint et al.	2014	Yes	Yes	No	Yes	No	Yes	Yes	No	No	No	Yes
Gopal et al.	2014	Yes	Yes	No	Yes	Yes	Yes	Yes	Yes	No	No	Yes
Slagt et al.	2015	Yes	Yes	No	No	Yes	Yes	Yes	Yes	No	Yes	Yes
Suehiro et al.	2015	Yes	Yes	Yes	Yes	No	Yes	Yes	Yes	No	Yes	Yes
Montenij et al.	2015	Yes	Yes	Yes	Yes	Yes	Yes	Yes	Yes	Yes	Yes	Yes
Broch et al.	2015	Yes	Yes	Yes	No	No	Yes	Yes	Yes	Yes	Yes	Yes
Monnet et al.	2015	Yes	Yes	No	No	Yes	Yes	Yes	Yes	No	Yes	Yes
Shih et al.	2016	Yes	Yes	Yes	No	No	Yes	Yes	Yes	No	No	Yes
Eiferman et al.	2016	Yes	Yes	No	No	No	Yes	Yes	No	No	No	Yes
Grensemann et al.	2016	Yes	Yes	No	Yes	Yes	Yes	Yes	Yes	No	No	Yes
Montenij et al	2016	Yes	Yes	Yes	Yes	Yes	Yes	Yes	Yes	Yes	Yes	Yes
Ganter et al	2016	Yes	Yes	Yes	Yes	No	Yes	Yes	Yes	Yes	Yes	Yes

Study	Year	Measurement protocol described	Appropriate reference method	Predefined acceptable error (e.g., 30%)	Sample size justification	Normality check	Bland-Altman analysis	Mean difference + 95%-limits of agreement	Percentage error	Confidence intervals reported	Corrected for repeated measurements	Proportional bias assessment
Tomasi et al.	2016	Yes	Yes	Yes	No	No	Yes	Yes	Yes	No	Yes	No
Terada et al.	2016	Yes	Yes	Yes	No	No	Yes	Yes	Yes	No	Yes	Yes
Cho et al.	2016	Yes	Yes	Yes	No	Yes	Yes	Yes	Yes	No	Yes	Yes
van Drumpt et al.	2017	Yes	Yes	Yes	No	Yes	Yes	Yes	Yes	No	Yes	Yes
Grensemann et al	2017	Yes	Yes	No	No	Yes	Yes	Yes	Yes	No	Yes	Yes
Asamoto et al	2017	Yes	Yes	No	Yes	No	Yes	Yes	Yes	Yes	Yes	Yes
Biais et al	2017	Yes	Yes	Yes	Yes	Yes	Yes	Yes	Yes	No	Yes	Yes
Lee et al.	2017	Yes	Yes	Yes	No	Yes	Yes	Yes	Yes	No	Yes	No
Antal et al.	2017	Yes	Yes	Yes	No	Yes	Yes	Yes	Yes	No	No	Yes
Lamia et al.	2018	Yes	Yes	No	No	No	Yes	Yes	Yes	No	No	Yes
Lin et al.	2018	Yes	Yes	Yes	No	No	Yes	Yes	Yes	No	Yes	Yes
Geisen et al.	2018	Yes	Yes	Yes	No	No	Yes	Yes	Yes	No	Yes	Yes
Eisenried et al.	2019	Yes	Yes	No	No	No	Yes	Yes	Yes	No	Yes	Yes
Kim et al.	2019	Yes	Yes	Yes	No	Yes	Yes	Yes	Yes	No	No	No

Study	Year	Measurement protocol described	Appropriate reference method	Predefined acceptable error (e.g., 30%)	Sample size justification	Normality check	Bland-Altman analysis	Mean difference + 95%-limits of agreement	Percentage error	Confidence intervals reported	Corrected for repeated measurements	Proportional bias assessment
Maeda et al.	2019	Yes	Yes	Yes	No	No	Yes	Yes	Yes	No	No	Yes
Kusaka et al.	2019	Yes	Yes	Yes	Yes	Yes	Yes	Yes	Yes	Yes	Yes	Yes
Khwannimit et al.	2020	Yes	Yes	Yes	No	Yes	Yes	Yes	Yes	No	Yes	Yes
Saugel et al.	2020	Yes	Yes	No	No	No	Yes	Yes	Yes	No	Yes	Yes
Greiwe et al.	2020	Yes	Yes	No	No	No	Yes	Yes	Yes	No	Yes	Yes
Greiwe et al.	2020	Yes	Yes	No	No	No	Yes	Yes	Yes	No	Yes	Yes
Heijne et al.	2021	Yes	Yes	Yes	Yes	No	Yes	Yes	Yes	Yes	Yes	Yes
Milam et al.	2021	Yes	Yes	No	No	No	Yes	Yes	Yes	No	No	Yes
Persona et al.	2021	Yes	Yes	Yes	Yes	Yes	Yes	Yes	Yes	No	No	Yes
Mukkamala et al.	2021	Yes	Yes	No	No	No	Yes	Yes	No	No	Yes	Yes
Oh et al.	2022	Yes	Yes	Yes	No	No	Yes	Yes	Yes	Yes	Yes	Yes
Ylikauma et al.	2022	Yes	Yes	Yes	No	No	Yes	Yes	Yes	Yes	Yes	Yes
Ylikauma et al.	2022	Yes	Yes	No	No	No	Yes	Yes	Yes	Yes	Yes	Yes
Halemani et al.	2022	Yes	Yes	Yes	Yes	No	Yes	Yes	Yes	Yes	Yes	Yes

Study	Year	Measurement protocol described	Appropriate reference method	Predefined acceptable error (e.g., 30%)	Sample size justification	Normality check	Bland-Altman analysis	Mean difference + 95%-limits of agreement	Percentage error	Confidence intervals reported	Corrected for repeated measurements	Proportional bias assessment
Murata et al.	2022	Yes	Yes	Yes	Yes	No	Yes	Yes	Yes	No	Yes	Yes
Kee et al.	2023	Yes	Yes	No	Yes	No	Yes	Yes	Yes	No	Yes	Yes
Khanna et al.	2023	Yes	Yes	No	No	No	Yes	Yes	Yes	No	Yes	Yes
Wu et al.	2023	Yes	Yes	Yes	No	No	Yes	Yes	Yes	No	No	No
Ordoñez-Rufat et al.	2023	Yes	Yes	No	No	Yes	Yes	Yes	Yes	No	Yes	Yes
Takei et al.	2023	Yes	Yes	Yes	Yes	No	Yes	Yes	Yes	Yes	No	Yes
Ylikauma et al.	2023	Yes	Yes	Yes	Yes	No	Yes	Yes	Yes	Yes	Yes	Yes
Ronkainen et al.	2024	Yes	Yes	Yes	Yes	No	Yes	Yes	Yes	Yes	Yes	Yes
Dinesen et al.	2025	Yes	Yes	Yes	Yes	No	Yes	Yes	Yes	Yes	Yes	Yes
